# Influence of genotype and soil on specialized metabolites production and bacterial microbiota associated to wild hop (*Humulus lupulus* L.): an early-stage study

**DOI:** 10.3389/fpls.2025.1702956

**Published:** 2025-10-21

**Authors:** Florent Ducrocq, Omar Hafidi, Jérémy Grosjean, Alain Hehn, Séverine Piutti

**Affiliations:** Université de Lorraine, INRAE, LAE, Nancy, France

**Keywords:** *Humulus lupulus* (L.), wild hops, metabolome, bacterial communities, roots, leaves, genotype×soil interaction

## Abstract

Hop (*Humulus lupulus* L.) is a dioecious climbing plant that is emblematic for the brewing industry because of its specialized metabolites. Many studies have focused on hop metabolism without considering the microbiota associated with hop tissues, although over the past decade, a paradigm shift has redefined plants as holobionts, with complex associations between the plant host and its associated microbial communities. In this study, we investigated the effects of three wild hop genotypes cultivated in two different agricultural soils under controlled conditions on specialized metabolite production and on bacterial community composition across different hop compartments (rhizosphere soil, roots, and leaves). Phytochemical analysis of leaf contents revealed distinct metabolic profiles across the six ‘genotype×soil’ interactions, driven by variations in the biosynthesis of prenylated chalcones, α- and β-type bitter acids, and their derivatives. PERMANOVA results demonstrated that both ‘genotype’ and ‘soil’ factors significantly influenced leaf metabolite composition, each explaining approximately 28% of the observed variance. However, the strongest effect was observed for the ‘genotype×soil’ interaction, which accounted for 66% of the variance. In parallel, soil type, hop genotype, and their interaction significantly shape hop-associated bacterial communities, with a predominant interaction effect in each compartment (rhizosphere soil, roots and leaves) (R² = 0.74, 0.74 and 0.32, respectively). Furthermore, Spearman microbiome–metabolome correlation analysis revealed that bacterial families were positively correlated with the biosynthesis of key metabolites, particularly bitter acids. Our findings further suggest that the hop-associated microbiota may contribute to metabolic biosynthesis, opening new perspectives for optimizing metabolite biosynthesis through microbiome manipulation.

## 1 Introduction

*Humulus lupulus* L. stands as an emblematic industrial crop in the French Northeast Region. Although traditionally valued for its medicinal properties (Zanoli and Zavatti, 2008), its primary modern application lies in the brewing industry. The attractiveness of hops in both contexts is due to the specialized metabolites produced in the lupulin glands, including bitter acids and polyphenols. The biosynthesis of these metabolites is influenced by several factors, including hop genotype (De Cooman et al., 1998) and environmental factors (De Keukeleire et al., 2007; Pistelli et al., 2018; Kunej et al., 2020), resulting in genotype×environment interactions. Among environmental parameters, soil properties and climatic conditions have already been demonstrated to affect specialized metabolite biosynthesis in hops, causing metabolic variations, as has been shown for the Amarillo (Van Holle et al., 2017) and Comet (Rosa et al., 2025) cultivars. Moreover, soil appears to play a major role in hop quality. For instance, Ruggeri et al. (2023) reported that the Cascade cultivar achieved higher yields when grown on light-textured soils (Ruggeri et al., 2023). In addition, soil pH and the concentrations of zinc, sulfur, and manganese were shown to influence the production of certain specialized metabolites in Cascade and Mosaic cultivars in a genotype-dependent manner (Féchir et al., 2023). Even if wild hop genotypes can develop in different ecological habitats, suggesting adaptation to a range of pedoclimatic conditions, to our knowledge, no study has evaluated the effects of soil type on their metabolic content. As mentioned, factors such as soil porosity, pH, nutrient availability and soil organic mattercan affect plant development (Abdul Khalil et al., 2015). These soil characteristics also impact the structure and diversity of the microbial communities recruited by the plant (Tkacz et al., 2015). Indeed, the plant-associated microbiota has emerged as a key player that significantly influences plant health, growth, and metabolic functions (Müller et al., 2016).

In fact, over the past decade, a paradigm shift has redefined plants as holobionts, emphasizing their close association with microbial communities (Simon et al., 2019). Plants in their natural environments interact with diverse biological organisms, including mostly bacteria, fungi, oomycetes, algae, and archaea (Müller et al., 2016). This microbial colonization occurs across all plant compartments, from the rhizosphere to the root (rhizoplane) and leaf surfaces (phyllosphere), as well as inside the root and leaf tissues (endosphere). Accordingly, the microbiota can be categorized as rhizospheric (root-adhering soil), epiphytic (living on the surface of the organs) or endophytic (living inside the tissues) (Bulgarelli et al., 2013). The microbial community in each compartment is shaped by plant characteristics and environmental factors. For example, rhizosphere and root endosphere microbial communities are strongly influenced by soil physicochemical properties such as pH, water, and nutrient bioavailability (Custódio et al., 2022). In contrast, phyllosphere microbial communities are colonized primarily by airborne microorganisms, with weak contributions from soil and seeds (Gong and Xin, 2021), and are further modulated by abiotic factors such as UV radiation, precipitation and biotic interactions with herbivores and pollinators. Finally, the host plant itself actively selects its microbiota through mechanisms involving root exudates, specialized metabolites, immune responses, and cuticle width, which are influenced by the plant’s genotype (Müller et al., 2016; Pascale et al., 2020; Brown et al., 2020). In line with this selective process, microbiota diversity decreases along the gradient from soil to leaves, ranging from 10^6–^10^9^ CFU/g of soil, from 10^4–^10^8^ CFU/g of root, and from 10^6–^10^7^ cells per cm² of leaf surface (Bulgarelli et al., 2013). These selective processes are also dynamic and vary according to the plant’s developmental stage (Comeau et al., 2020).

Despite progress in understanding plant–microbe interactions, the microbiota associated with hops remains poorly explored, despite its economic importance in the brewing industry. Notable exceptions include a study by Allen and collaborators that characterized the microbial communities of hop flowers from several varieties cultivated at the field scale. Among the most abundant taxa, Proteobacteria were predominant, with *Pseudomonas* and *Sphingomonas* emerging as the most representative genera (Allen et al., 2019). However, there is limited knowledge about the structure and diversity of the microbiota in other hop compartments and its potential influence on the hop metabolome.

In this study, we investigated the bacterial communities associated with three wild hop genotypes from northeastern France, which were previously selected for their contrasting metabolic profiles (Ducrocq et al., 2025). These wild genotypes were cultivated on two different agricultural soils under controlled environmental conditions. We characterized the bacterial communities in three compartments: rhizosphere soil, roots (epiphytic and endophytic microbiota), and leaves (endophytic microbiota) at the vegetative stage. Additionally, we analyzed the metabolic contents of the leaves of these wild genotypes. Our objectives were to evaluate how soil type and hop genotype influence bacterial communities across different compartments and to explore whether variations in associated taxa are correlated with specialized metabolite production in leaves. Specifically, we hypothesized that (i) the accumulation of specialized metabolites in hop plants is modulated by both genotype and soil factors, with genotype exerting a predominant influence; (ii) each of the four studied compartments harbors a distinct and specific bacterial community, with the soil type strongly shaping rhizosphere and root communities, whereas the hop genotype predominantly shapes endophytic communities; and (iii) specific bacterial taxa are associated with specialized metabolite accumulation in leaves.

## 2 Materials and methods

### 2.1 Wild hop genotypes, experimental design, and management

According to reference (Ducrocq et al., 2025), exclusively wild hops (*Humulus lupulus* var. *lupulus*) were randomly collected in various ecological habitats (forest edges, hedges, riparian zones, and field margins), following their natural distribution (range of 30km from Nancy). Wild hops were collected in accordance with the rules of Nagoya Protocol and the French Biodiversity law (decision issued by the Ministry of Ecological and Territorial Cohesion, ATDL2500141S/916). A section of the main stem was collected for each plant. Aeroponic cuttings were prepared from these stem segments to induce root development, prior to their transfer into potting soil. From this collection, three wild hop genotypes (G3, G27 and G31) were selected for this experiment based on their contrasting metabolic profiles. The “parent” wild hops were maintained in a greenhouse, in potting soil adhering to organic farming conditions (no pesticides and no synthetic fertilizers), and experienced natural temperature and light fluctuations from March until July 2023. Aeroponic cuttings (n = 32) were performed from the ‘parent’ hop’s main stem for each genotype to constitute biological replicates (clones). Soils were sampled from two different experimental farms: the Arvalis station at Saint-Hilaire-en-Woëvre (France) (soil A) and the Bouzule farm at Laneuvelotte (France) (soil B) in the field plot (wheat as the preceding crop) (within the top 20cm) and sieved at 5mm. The soils were then prepared by mixing them separately with sand at 70/30 (soil/sand, (v/v)) to increase the soil porosity and improve root sampling at harvest. The physicochemical properties of the samples were determined by Celesta Lab (Mauguio, France) (bare soil: described in **Supplementary Table S1**). The two soils are very different from soil A, which corresponds to an acidic hydromorphic loamy soil, whereas soil B is a calcareous silty loamy soil. Each rooted clone cutting was placed in a 12 L plastic pot (280×240 mm) filled with prepared soil and watered at 70% WHC. The pots were then placed in a greenhouse from the end of August 2023 until the end of October 2023, with automatic watering to maintain 70% of the WHC throughout the experiment. Thus, two factors were tested: soil type (with two modalities: A and B) and hop genotype (with three modalities: G3, G27, and G31), resulting in a total of six experimental conditions for 96 pots.

### 2.2 Sample collection and data acquisition

#### 2.2.1 Harvesting of leaves, roots, stems, and rhizosphere soil

Four biological replicates per condition were selected based on their similar levels of development at the vegetative stage. At harvest time and for each replicate, we measured the main stem length and weighed the fresh shoot and root (without rhizome) masses. The five top leaves on each side were retained for metabolomic and bacterial analyses. For metabolomic analysis, leaves were directly frozen in liquid nitrogen. Otherwise, for metabarcoding analysis, a surface sterilization step was performed as follows: 2 minutes in 300 mL of 70% ethanol, 5 minutes in 300 mL of 1.2% sodium hypochlorite, and finally, 2 minutes× 3 washes in sterile distilled water (2× 300 mL and 1× 50 mL). Each step was performed on an agitator at 120 rpm (Orbital Shaker, Major Science, Saratoga, USA). The sterilized leaves were then dried with sterile paper before being frozen in liquid nitrogen. The final sterile water bath was kept in a 50 mL Falcon tube and was centrifuged at 5000×g for 10 minutes at 4 °C (BR4i, Jouan, France). The pellet was resuspended in 300 µL of sterile PBS (8.1 mM Na2HPO4, 1.76 mM KH_2_PO_4_, 2.7 mM KCl, and 137 mM NaCl, pH 7.4) and inoculated on 10% TSA petri dishes containing 100 mg.L^-1^ cycloheximide at 28 °C. Bacterial development was monitored for 5 days after incubation. Roots were carefully extracted from the pots, and rhizosphere soil was collected from each pot and stored in a 15 mL tube at −40 °C for subsequent metabarcoding analyses. The roots were then carefully washed with tap water to ensure that there was no more soil, dried with a paper towel, frozen in liquid nitrogen, and stored at −80 °C, as were the leaves and stems.

#### 2.2.2 Metabolomic profiling

All chemical solutions (methanol, acetonitrile, and formic acid) were obtained from the same supplier (Carlo Erba Reagents S.A.S., Val-de-Reuil, France).

#### 2.2.2.1 Extract preparation

Metabolite extraction and UHPLC-ESI-MS (ultra-high-performance liquid chromatography-electrospray ionization process-mass spectrometry) analysis were conducted as previously described (Ducrocq et al., 2025) with minor modifications. Briefly, leaves were ground using a mortar and pestle with liquid nitrogen. A double maceration extraction was performed on 100 mg (± 0.2 mg) of fresh leaf powder. Four milligrams of dry extract were solubilized at a 1/20 ratio (m/v) in 80% MeOH [MeOH/H_2_O (pure) 80/20 (v/v)]. Finally, 49 µL of each extract was mixed with 1 µL of taxifolin (6.574 mM – 100% MeOH) (MedChem Express HY-N0136, Thermo Fisher Scientific), which was used as an internal standard. Extracts were subsequently analyzed using UHPLC-ESI-MS.

##### 2.2.2.2 Molecular identification and statistical analysis

To profile the metabolites in our different hop extracts, the raw data files were uploaded into Compound Discoverer™ software (version 3.3) (Thermo Fisher Scientific, Bremen, Germany). Briefly, the software workflow included peak detection, chromatogram alignment, and peak grouping in features and raw data files based on blank, QC, and sample files. Each feature corresponds to a specific *m/z* at a given retention time. The compounds were identified through (i) elemental composition prediction; (ii) searching in mass/formula databases (including internal databases with commercial standards) and public databases [LOTUS: Natural Products, with “Humulus” search (https://lotus.naturalproducts.net/search/simple/Humulus)]; and (iii) with MS^2^ information, searching in-house and the public spectral databases mzCloud, and MoNA (https://mona.fiehnlab.ucdavis.edu/). Statistical analyses were performed with peak area data recovered by Compound Discoverer software for the main compounds identified in RStudio software (version 4.3.2) (R Core Team, 2024) using the packages ‘FactoMineR’ (version 2.11) (Lê et al., 2008), ‘factoextra’ (version 1.0.7) (Kassambara and Mundt, 2020), ‘corrplot’ (version 0.95) (Wei and Simko, 2024) (for PCA analyses), ‘gplots’ (version 3.2.0) (Warnes et al., 2024) and ‘RColorBrewer’ (version 1.1.3) (Neuwirth, 2022) (for heatmap analysis). Briefly, a PCA was performed to explore metabolic data to deduce the variability explained by the variables (targeted metabolites), on which axes to project data, the metabolites contributing the most to sample differentiation, and the spatial distribution of the samples. Moreover, a heatmap was generated to understand biologically why the samples are distributed in different ways. A permutation-based multivariate analysis of variance (PERMANOVA) was conducted (10,000 permutations) using the ‘vegan’ package (Oksanen et al., 2025) to assess the effects of soil, genotype, and their interaction on the identified specialized metabolites.

#### 2.2.3 Metabarcoding analysis

##### 2.2.3.1 DNA extraction and sequencing

The surface-sterilized leaves, roots, and rhizosphere soil were ground with a mortar and pestle with liquid nitrogen to crush and homogenize the samples. The genomic DNA (gDNA) of leaves and roots was extracted from 100 mg of the resulting powder using a DNeasy^®^ Plant Mini Kit (Qiagen, Hilden, Germany) and from 500 mg of rhizosphere soil using a FastDNA™ Spin Kit for Soil (MP Biomedicals, Eschwege, Germany), both of which were performed according to the manufacturer’s instructions. The quality and quantity of the DNA were checked using a NanoDrop One^C^ (Thermo Fisher Scientific, Madison, USA), and the DNA was stored at −20 °C. Total gDNA was sequenced using the bacterial V5 and V7 regions of 16S rDNA. Sample quality control, library preparation, barcode multiplexing, amplicon sequencing and demultiplexing of reads were performed by GenoScreen (Lille, France). Sequencing was performed via Illumina MiSeq with a 2×250 bp paired-end library.

##### 2.2.3.2 Statistical analyses and data processing

Statistical analyses were performed using RStudio software (version 4.3.2) (R Core Team, 2024). The comparisons of the hop morphological traits were conducted using the ‘stats’ package (version 4.3.2) with Shapiro, Bartlett, ANOVA2 and Tukey tests. The ‘DADA2’ package (version 1.30.0) (Callahan et al., 2016) was first used to process, align, and analyze the sequenced MiSeq reads. Briefly, reads were trimmed to maintain high-quality sequences using the filterAndTrim function and filtered with DADA2’s error simulation with the learnErrors function. Taxonomy was assigned to 16S rDNA with the SILVA reference database ‘silva_nr99_v138.1_train_set.fa’ (extracted from https://zenodo.org/records/4587955) (Quast et al., 2012). The bacterial taxonomy dataset was then cleaned by removing amplicon sequence variants (ASVs) with a taxonomic affiliation to chloroplasts and mitochondria. For further analysis, the dataset was split according to the different studied compartments and further subdivided based on soil type and hop genotype. For α diversity calculations, the datasets were first rarefied based on the lowest number of reads using the ‘phyloseq’ package (version 1.46.0) (McMurdie and Holmes, 2013). Several diversity indices (Shannon, Simpson, Chao1 and Observed) were calculated and visualized using the ‘ggplot2’ package (version 3.5.1) (Wickham, 2016). Kruskal–Wallis tests were then performed, followed by *post hoc* pairwise multiple comparisons (Dunn’s test) using the ‘rstatix’ (version 0.7.2) (Kassambara, 2023), ‘multcompView’ (version 0.1.10) (Graves et al., 2024), ‘tidyverse’ (version 2.0.0) (Wickham et al., 2019) and ‘FSA’ (version 0.9.6) (Ogle et al., 2025) packages. For β diversity calculations, datasets were normalized by transforming raw counts into relative abundances using the ‘phyloseq’ package. Nonmetric multidimensional scaling (NMDS) was performed using the ‘vegan’ (version 2.6.10) (Oksanen et al., 2025), ‘dplyr’ (version 1.1.4) (Wickham et al., 2023a) and ‘tidyr’ (version 1.3.1) (Wickham et al., 2024) packages, which are based on the Bray–Curtis distance, and visualized using ‘ggplot2’. Taxonomic abundance at the phylum level was visualized using the ‘ggplot2’ and ‘scales’ (version 1.3.0) (Wickham et al., 2023b) packages, which consider only taxa representing more than 1% of the total abundance. Permutation-based multivariate analysis of variance (PERMANOVA) was performed (10,000 permutations) to assess the effects of soil, genotype, and their interaction on bacterial communities using the ‘vegan’ package. Moreover, to identify taxa that have significant (LDA score > 2, FDR-adjusted p < 0.05) differential abundance across the six conditions (‘genotype×soil’), linear discriminant analysis effect size (LEfSe) analysis was performed on MicrobiomeAnalyst (Chong et al., 2020; Lu et al., 2023) at the “family” level. Finally, to investigate the core bacterial microbiota, we identified the ASVs shared within each compartment (rhizosphere soil, roots, and leaves) by comparing the same hop genotype grown in two different soils (A or B). Specifically, we compared the conditions “G3A vs. G3B”, “G27A vs. G27B”, and “G31A vs. G31B” for each compartment. The shared ASVs were then assigned to their respective taxonomic families. The common families identified in each condition were subsequently counted and cross-compared among the three conditions to determine the bacterial families consistently present in a given compartment (regardless of hop genotype and soil type). Finally, we constructed a pie chart using the “webr” package (version 0.1.5) (Moon, 2020) to visualize the common bacterial families in each compartment.

#### 2.2.4 Integrating metabarcoding and metabolomic data

To integrate the two datasets, we selected the bacterial families that were previously identified as significantly differentially abundant in the rhizosphere soil and root compartments via LEfSe analysis. Their relative abundances in each sample were used alongside the peak area data of the main identified specialized metabolites. Spearman’s correlation coefficients were calculated to assess significant (p < 0.001) positive and negative correlations between bacterial families and specialized metabolites identified using the ‘corrplot’ package (version 0.95).

## 3 Results

### 3.1 Morphological traits of wild hops assessed at harvest

Four biological replicates were used per condition at the vegetative stage. Morphological traits were measured and are reported in **Supplementary Figures S1A, B**, with the statistical tests summarized in **Table 1**.

**Table 1:** Two-way ANOVA and Tukey *post hoc* test results for the morphological traits of wild hops at harvest.

		Variables		
		Stem size (cm)	Fresh shoot mass (g)	Fresh root mass (without rhizome) (g)
G3 (mean ± sd)		66 ± 14.25	3.76 ± 1.10	9.18 ± 3.64
G27 (mean ± sd)		43.88 ± 8.38	2.65 ± 0.66	3.71 ± 1.77
G31 (mean ± sd)		58.63 ± 10.03	3.63 ± 0.65	7.64 ± 2.05
Two-way ANOVA factors		Results		
Pr(>F) for ‘Soil’		0.1221	0.2420	0.1053
Pr(>F) for ‘Genotype’		0.0196 (*)	0.1160	0.0063 (*)
Pr(>F) for ‘Genotype×Soil’		0.8648	0.6300	0.1759
Tukey (*post hoc* comparisons)		Results		
Soil	p.adj B-A	X	X	X
Genotype	p.adj 27-3	0.0167 (*)	X	0.0059 (*)
	p.adj 31-3	0.5691	X	0.5844
	p.adj 31-27	0.1274	X	0.0482 (*)
Genotype×Soil	X	X	X	X

Significant values are shown in bold text, followed by (*).

These results indicate that, compared with genotypes 3 and 31, genotype 27 has shorter stems (**Supplementary Figure S1A**) and a lower fresh rooting system weight (**Supplementary Figure S1B**). This phenomenon appears to be a genotype effect on hop morphological development. Among the three analyzed morphological traits, the ‘soil’ and ‘genotype×soil’ factors had no significant effect on each variable, whereas the ‘genotype’ factor had a significant effect on the ‘stem size’ and ‘fresh root mass’ variables (*p*=0.0196 and 0.0063, respectively). For the ‘stem size’ variable, we observed a significant difference when comparing genotypes 3 and 27 (*p*=0.0167). For the ‘fresh root mass’ variable, a significant difference was found between genotypes 3 and 27 (*p*=0.0059) and between genotypes 27 and 31 (*p*=0.0482) (**Table 1**).

### 3.2 Influence of soil and genotype on specialized metabolite production in wild hop leaves

Phytochemical analyses led to the identification of twelve metabolites in the extracts: cohulupone, hulupone, adhulupone, xanthohumol, humilinic acid, cohumulone, humulone + adhumulone, desoxyhumulone, postlupulone, lupulone E, colupulone and lupulone + adlupulone. The molecules identified were β-type bitter acids (co, n-, and ad-lupulone). In addition to bitter acids, oxidized derivatives of β-acids, namely, co, n- and ad-hulupone, were identified. Moreover, we also identified xanthohumol, a well-known flavonoid in hops. Finally, other molecules derived from bitter acids, such as postlupulone, lupulone E (from β-acids), and desoxyhumulone (from α-acids), were identified (**Supplementary Figure S2**).

**Figure 1:**
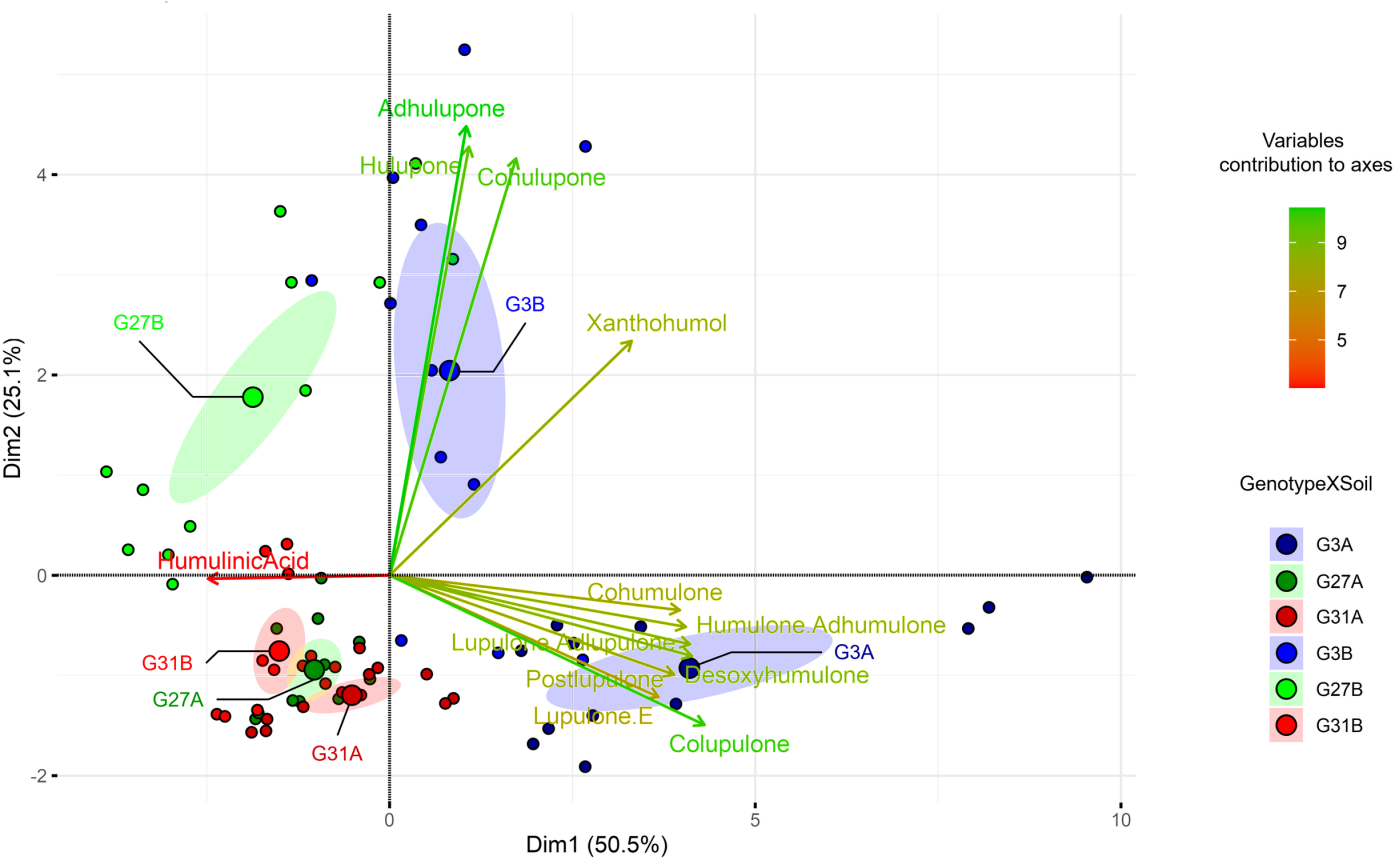
Principal component analysis (PCA) biplots based on hop leaf content according to the six studied conditions. The variable contributions to axes are shown in the gradient from red (low) to green (high). For conditions, the letter indicates the soil, and the number represents the hop genotype.

To assess the metabolic diversity among the wild hop leaves under the six conditions, we performed a multivariate analysis. The PCA biplot we obtained (**Figure 1**) clearly revealed metabolic differences among the six conditions, highlighting their distinct metabolic profiles.

**Figure 2:**
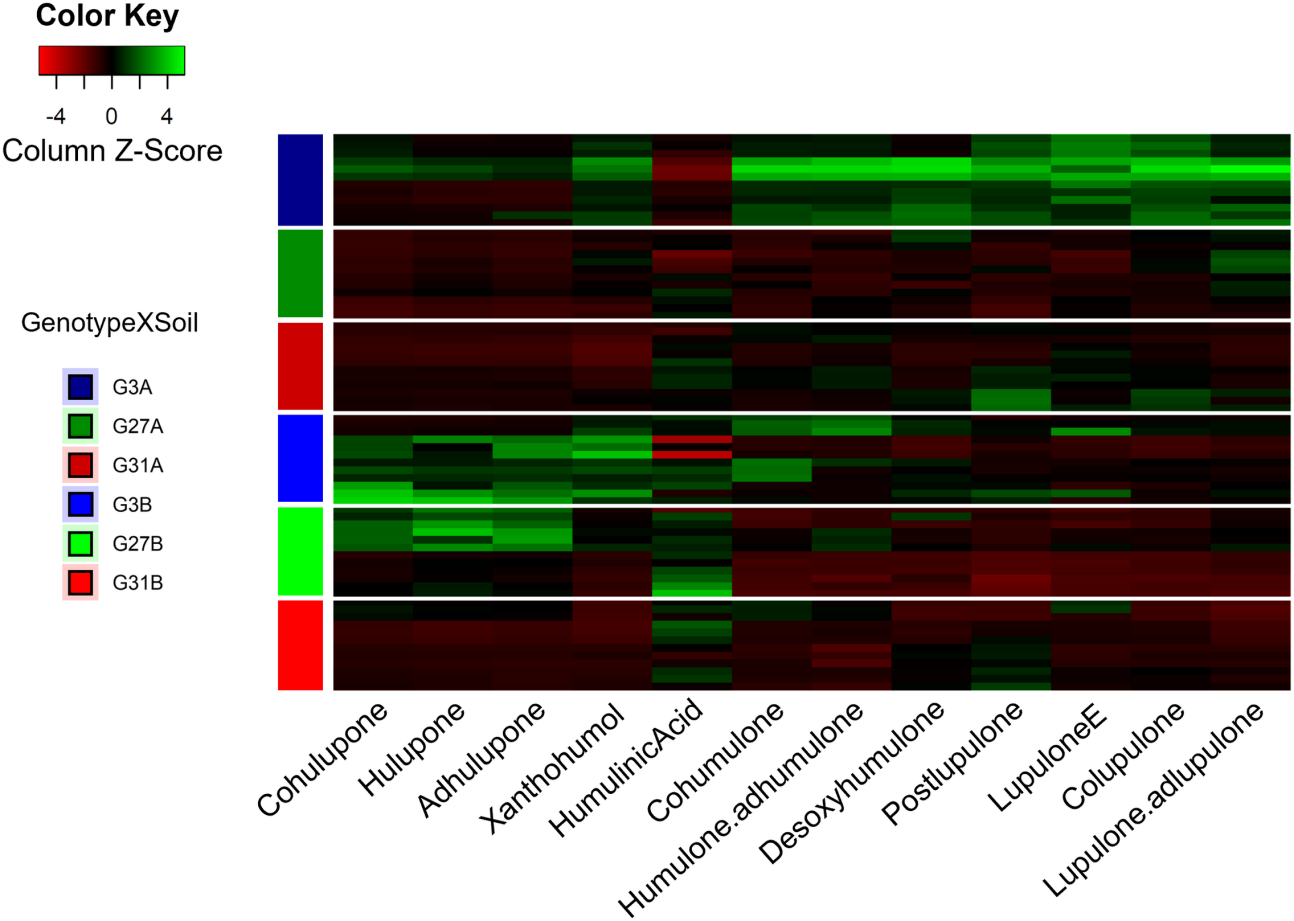
Heatmap of the metabolic contents of hop leaves according to the six studied modalities. The metabolite production is shown in the gradient from red (low) to green (high). For conditions, the letter indicates the soil, and the number represents the hop genotype.

Considering the twelve specialized metabolites identified, 76.6% of the observed variance was explained (PC1 50.5%, PC2 25.1%). The predominant specialized metabolites explaining the greatest difference between conditions are β-type bitter acids (colupulone) and β-type oxidized derivatives (cohulupone, hulupone, adhulupone). Axis 1 is predominantly associated with bitter acids (types α and β) and their derivatives (desoxyhumulone, postlupulone, and lupulone E) on the positive side, whereas humulinic acid is associated with the negative coordinates of this axis. Conversely, positive coordinates on Axis 2 appear to be driven by β-type oxidized derivatives, as well as xanthohumol. Genotype 3 cultivated on soil A (G3A condition) presented positive coordinates on Axis 1, whereas the same genotype cultivated on soil B (G3B condition) presented positive coordinates on Axis 2, suggesting that these soil types led to the enrichment of different metabolites. The G3A modality resulted in increased production of bitter acids (α- and β-type) along with related compounds such as desoxyhumulone, postlupulone, and lupulone E. In contrast, the G3B modality resulted in increased levels of β-type oxidized derivatives and xanthohumol (**Figure 2**).

In contrast, genotype 31, regardless of the soil type (G31A and G31B), displays negative coordinates on both axes in the same way as does genotype 27 cultivated on soil A (G27A), indicating that they are deficient in the identified specialized metabolites. They presented lower overall production of specialized metabolites and a similar chemical profile (**Figure 2**), explaining their close clustering in the PCA (**Figure 1**). Finally, genotype 27 cultivated on soil B (G27B) appears to be intermediate between the metabolically rich conditions (G3A, G3B) and the poorer ones (G31A, G31B, and G27A), as it is positively associated with Axis 2 and negatively associated with Axis 1, suggesting that this condition is enriched in β-type oxidized derivatives (cohulupone, hulupone and adhulupone) and in humulinic acid. Furthermore, for a given genotype, soil appears to influence the production of specialized metabolites, as evidenced by distinct chemical signatures. This effect is particularly pronounced in genotype 3, where plants grown in soil A (G3A) exhibit increased production of bitter acids (both α and β types), desoxyhumulone, postlupulone and lupulone E, whereas those grown in soil B (G3B) predominantly produce β-type oxidized derivatives. Finally, compared with our previous study (Ducrocq et al., 2025), our results confirm that G3 is a high producer, G27 is a moderate producer, and G31 is a low producer of specialized metabolites.

In addition, the specialized metabolites identified previously were significantly influenced by both variables (soil and genotype) according to the PERMANOVA results (data not shown). Individually, the soil and genotype had significant (*p*<0.001) and similar effects, each explaining approximately 28% of the variability in the leaf metabolic content (R² = 0.28 and 0.28, respectively). However, when considered together, the interaction between soil and genotype (genotype×soil) also had a significant effect (*p*<0.001), accounting for approximately 66% (R² = 0.66) of the variability in the metabolic content of the leaves. These results suggest a pronounced interaction between the soil type and hop genotype, which strongly influences specialized metabolite production.

### 3.3 Wild hop microbiome sequenced reads

Microbial diversity and community structure were investigated in the rhizosphere soil, roots, and leaves of three wild hop accessions cultivated in two different soils. Illumina MiSeq sequencing generated a total of 4,240,746 paired-end raw reads, with the number of reads per sample ranging from 10,582–112,413 (mean: 53,009). Across the three studied compartments, the average read count was highest for the rhizosphere soil samples (mean: 93,444; range: 78,376–112,413), followed by the root samples (mean: 34,584; range: 18,320–51,206) and the leaf samples (mean: 17,521; range: 10,582–37,577). Filtering, trimming, quality control, and chimera removal (~22% of reads) via the DADA2 pipeline (**Supplementary Table S2**) resulted in 8209 bacterial ASVs. We also removed 940 ASVs (7269 cleared ASVs in total) from the bacterial dataset because they matched mitochondrial or chloroplast sequences.

### 3.4 Richness of the microbiome of wild hops: α diversity

**Figure 3:**
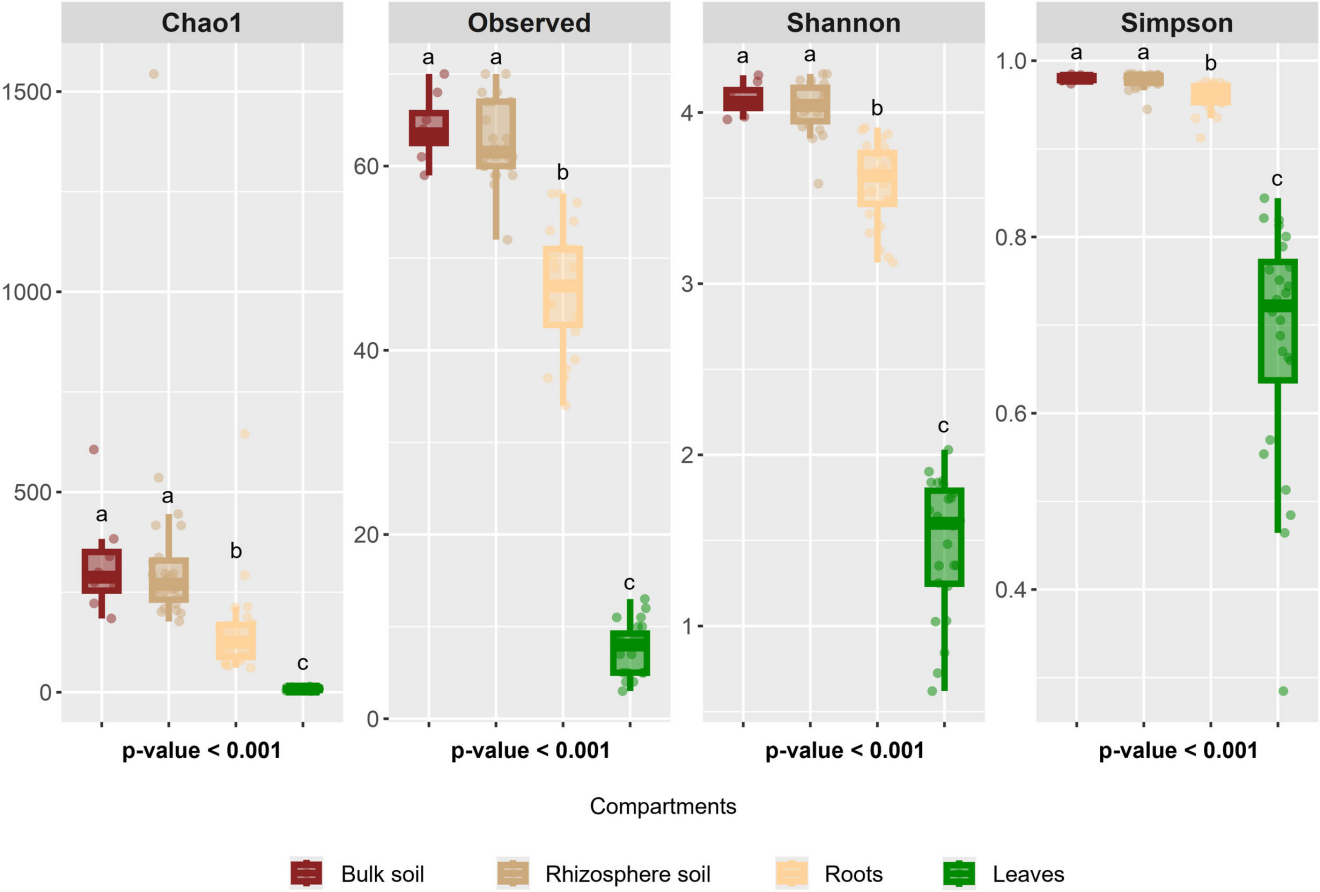
α-diversity indices across the studied compartments. Kruskal–Wallis test p values indicate significant differences, and different letters denote significant differences based on *post hoc* Dunn’s tests.

To assess how microbial communities are structured across different compartments, we first investigated whether each of the four studied compartments harbors a distinct and specific microbiota. Additionally, we examined the respective influences of soil type and hop genotype, hypothesizing that soil type would have a stronger impact on the rhizosphere microbiota, whereas plant genotype would play a predominant role in shaping the bacterial communities associated with roots and leaves. Alpha diversity metrics, including the Chao1, Observed, Shannon, and Simpson indices, revealed significant differences (*p*<0.001) across compartments, highlighting differences in bacterial richness, except for those in bulk soil and rhizosphere soil, which have the same α diversity. The results indicate that bacterial richness is highest in bulk soil (Chao1, 321.80 ± 90.88; Shannon, 4.08 ± 0.07) and rhizosphere soil (Chao1, 341.15 ± 137.73; Shannon, 4.03 ± 0.11), followed by roots (Chao 1, 152.48 ± 69.85; Shannon, 3.58 ± 0.19), whereas leaves present the lowest richness (Chao 1, 8.39 ± 2.53; Shannon, 1.47 ± 0.33) (**Figure 3**), suggesting a filtering effect within hop-associated compartments, progressively shaping bacterial communities.

To further explore α diversity, the dataset was divided according to compartment and further subdivided based on soil type and hop genotype. We first examined the effect on soil by comparing the same genotype grown in different soils. In the rhizosphere soil, the Simpson index was significantly influenced by the soil type when the same genotype cultivated in soil A or soil B was compared (G3A vs. G3B, G27A vs. G27B, and G31A vs. G31B; *p*=0.021) (**Supplementary Figure S3A**). For all the genotypes, the bacterial richness was greater in the rhizosphere of the plants grown in soil A. In the root compartment, a significant soil effect was also detected (*p*=0.021) (**Supplementary Figure S3B**). Interestingly, although bacterial richness was greater in the rhizosphere of the plants grown in soil A, the root-associated bacterial communities presented greater richness in the genotypes cultivated in soil B. If the hop actively recruits beneficial bacteria within its tissues, this result suggests that soil B may harbor a native bacterial community that is more beneficial to hops or more adapted to hop root conditions than soil A is. In contrast, in the leaf compartment, the soil type had no significant effect on the Simpson index when the Simpson index was compared across the same genotype (**Supplementary Figure S3C**).

Conversely, we analyzed the genotype effect within each soil type. When each soil type was examined separately, no significant genotype effect was detected in the rhizosphere soil (**Supplementary Figure S4A**) or leaf compartments (**Supplementary Figure S4C**). However, a significant effect was detected in the root compartment, where genotype 31 presented greater bacterial richness than did genotype 27 (*p*=0.049) (**Supplementary Figure S4B**). Additionally, bacterial community recruitment appeared to be both genotype- and soil-dependent, as no single genotype consistently displayed either high or low bacterial richness across all compartments and soil conditions. For example, in the rhizosphere soil of soil A, genotype 31 presented the highest richness, whereas in the same compartment of soil B, it presented the lowest richness (**Supplementary Figure S4A**). In the root compartment, genotype 31 consistently presented the highest richness in both soils A and B (**Supplementary Figure S4B**), whereas in the leaf compartment, it presented the lowest richness regardless of the soil type (**Supplementary Figure S4C**).

### 3.5 Diversity of the microbiome of wild hops: β diversity

**Figure 4:**
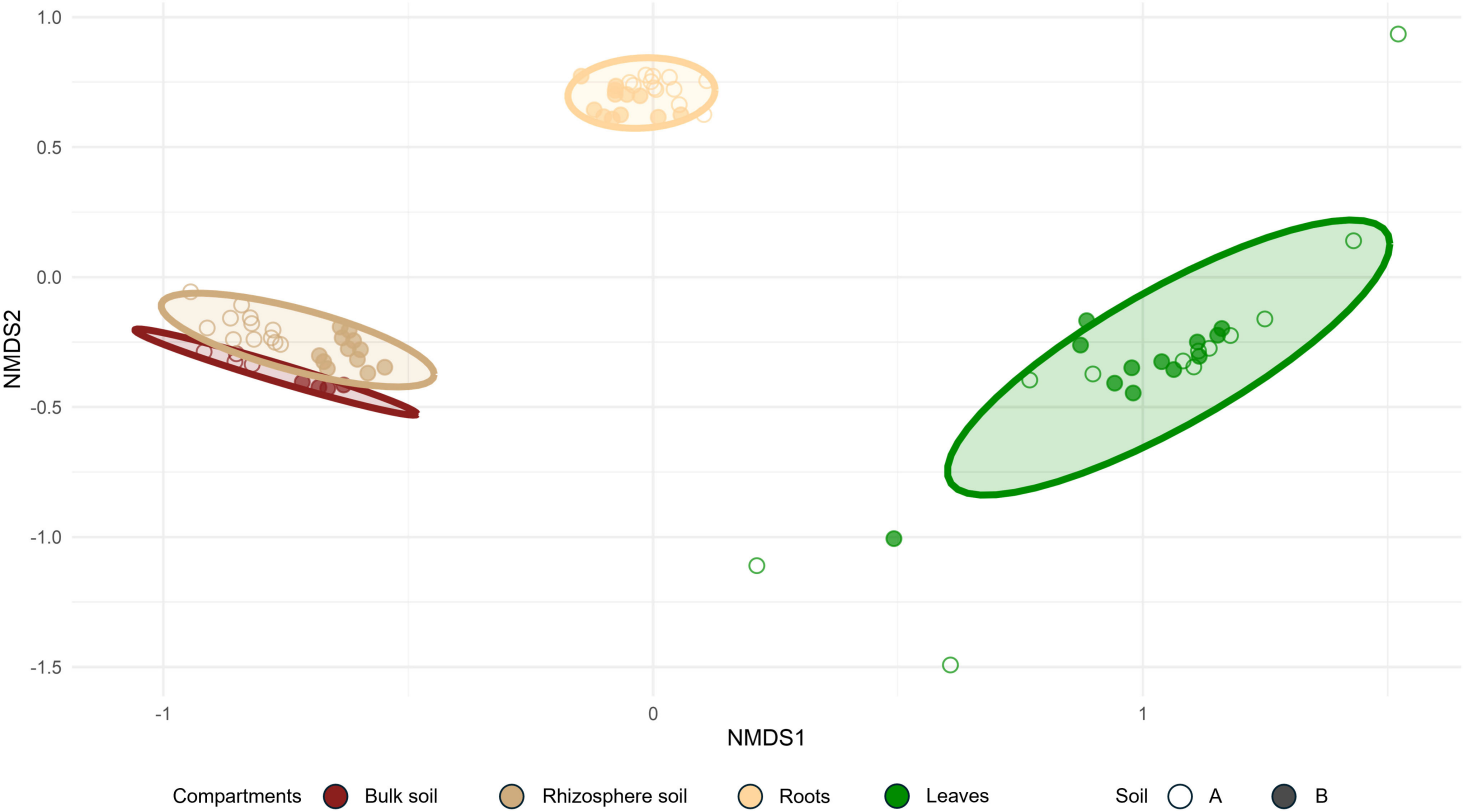
Bray–Curtis NMDS ordination of bacterial communities according to the compartments and soil type (stress = 0.08).

Beta diversity analysis confirmed that each of the four studied compartments harbored a distinct bacterial community. However, the bacterial compositions of the bulk soil and rhizosphere soil were similar (**Figure 4**).

Soil type had a strong influence on the bacterial communities in both the bulk and rhizosphere soil compartments, as evidenced by the clear separation between soil A and soil B, with samples from soil A displaying more negative coordinates. Nevertheless, this effect appeared weaker in the roots and even greater in the leaf compartments (**Figure 4**). This suggests that additional factors, beyond the soil type, contribute to the shaping of bacterial communities in hop-associated compartments, particularly in internal tissues.

**Figure 5:**
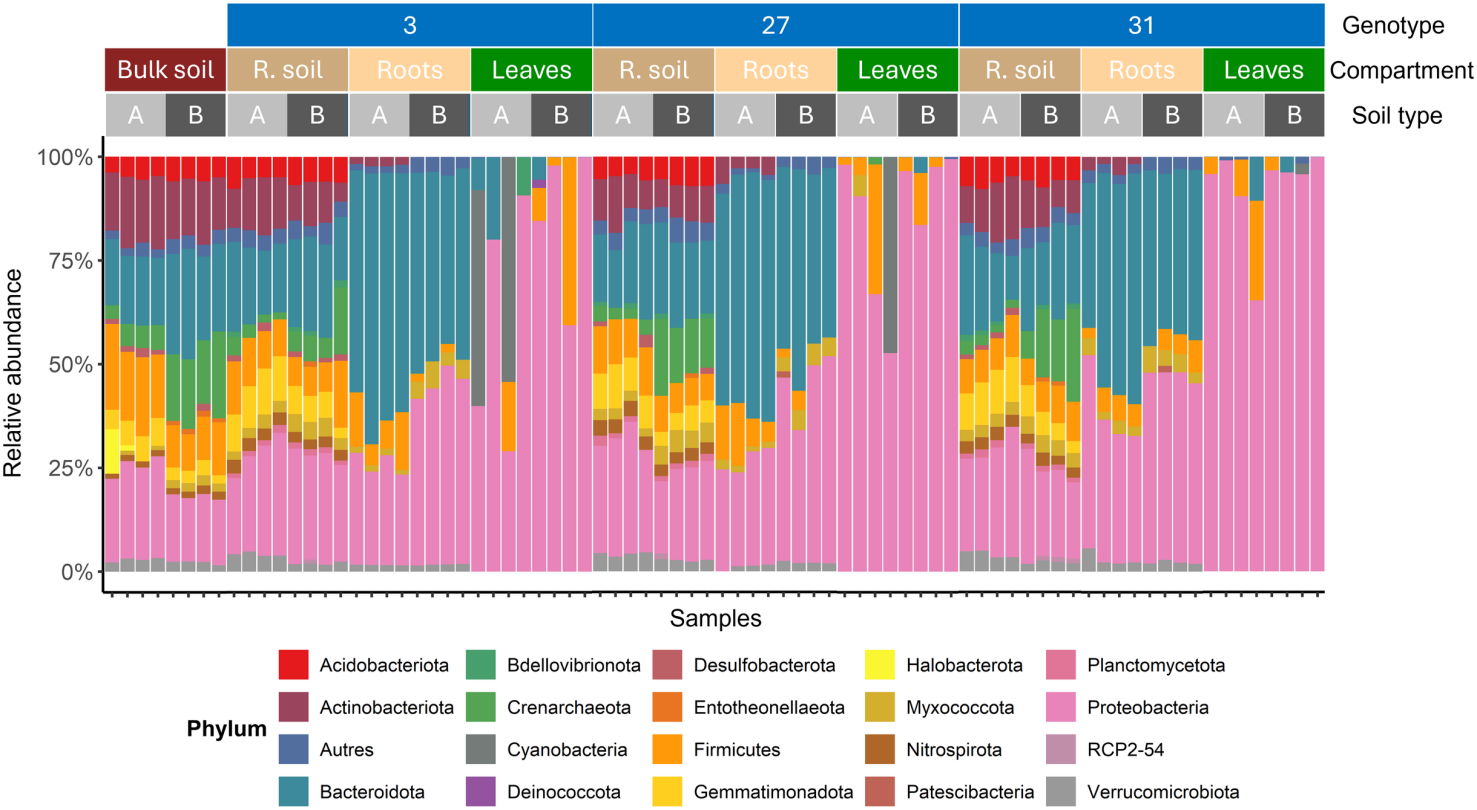
Bacterial community composition across samples according to the wild hop genotype, compartment and soil studied. The taxonomic composition is shown at the phylum level. Only phyla with a relative abundance greater than 1% across all samples were retained for visualization.

This compartment effect on the hop microbiota was also evident when the relative abundance of bacterial communities at the phylum level was examined (**Figure 5**).

Bulk soils A and B harbored distinct native bacterial communities (**Figure 5**), which influenced the recruitment of hop-associated microbiota. For example, soil B harbored a native bacterial microbiota with high relative abundances of Crenarchaeota, Bacteroidota, and Entotheonellaeota. In contrast, it presented lower levels of Firmicutes and lacked Halobacterota. These native differences in the bulk soil microbiota had downstream consequences for microbial recruitment in other compartments. Notably, both the rhizosphere and root microbiota differed between soil A and soil B for each genotype. In contrast, microbial patterns in the leaf compartment were more difficult to interpret than those in the other compartments because of greater heterogeneity within the same condition (‘genotype×soil’). Nevertheless, clear distinctions were observed between the microbiota of the rhizosphere soil, roots, and leaves, with a progressive increase in the relative abundance of the Proteobacteria phylum, highlighting compartment-specific recruitment. The previously suggested ‘filtering effect’ between the external and more internal compartments is obvious in this figure. However, as previously noted, the bacterial communities in rhizosphere and bulk soils appear relatively similar, sharing the same dominant phyla but with differing relative abundances. For example, the rhizosphere soil presented increased abundances of Proteobacteria, Planctomycetota, Nitrospirota, Myxococcota, Gemmatimonadota, and Bdellovibrionota. In contrast, the relative abundances of Entotheonellaeota, Firmicutes, and Crenarchaeota were decreased in the rhizosphere soil compared with those in the bulk soil. Although the root microbiota shared phyla with rhizosphere soil (Bacteroidota, Firmicutes, Myxococcota and Proteobacteria), several phyla were no longer present in hop roots, including Nitrospirota, Gemmatimonadota, Crenarchaeota, Bdellovibrionata, and Acidobacteriota. Additionally, despite being detected in both bulk soil and rhizosphere soil, Actinobacteriota were absent in the roots of all three genotypes grown in soil B, whereas they remained present in soil A for each genotype.

To further explore these patterns, each compartment was analyzed separately, considering both the soil type and hop genotype (**Table 2**). First, the observed differences in taxonomic richness across compartments once again suggest a filtering effect, with 5,195 taxa identified in the rhizosphere soil: 1,1884 in the roots and only 118 in the leaves.

**Table 2:** PERMANOVA results of soil, genotype, and interaction effects on hop compartment bacterial community structure.

	Variables	R²	p value
RHIZOPHERE SOIL (5195 taxa)	Soil	0.67237	< 0.001 (***)
	Genotype	0.03391	0.887
	Genotype×Soil	0.73903	< 0.001 (***)
ROOTS (1884 taxa)	Soil	0.59514	< 0.001 (***)
	Genotype	0.06916	0.458
	Genotype×Soil	0.73816	< 0.001 (***)
LEAVES (118 taxa)	Soil	0.0782	0.0533
	Genotype	0.1513	0.021 (*)
	Genotype×Soil	0.32118	0.012 (*)

p-value < 0.05 (*) ; p-value < 0.001 (***).

In the rhizosphere soil, PERMANOVA revealed a strong and significant effect of ‘soil type’ (R² ~67%, *p*<0.001), whereas ‘genotype’ had no significant impact. However, the strongest effect was observed for the ‘genotype×soil’ interaction (R² ~74%, *p*<0.001), indicating that the bacterial community composition in the rhizosphere is shaped by the combined influence of both factors. A similar pattern was observed in the root compartment, where ‘soil type’ had a strong influence on bacterial composition (R² ~60%, *p*<0.001), whereas ‘genotype’ had no significant effect. Once again, the most pronounced impact was observed for the ‘genotype×soil’ interaction (R² ~74%, *p*<0.001), suggesting that both factors together play crucial roles in shaping root-associated microbial communities. Finally, in the leaf compartment, the ‘soil’ effect was no longer significant. Instead, both ‘genotype’ and the ‘genotype×soil’ interaction significantly influenced bacterial composition (*p*=0.021 and *p*=0.012, respectively), although their explanatory power was lower than those of the rhizosphere and root compartments (R² ~15% and R² ~32%, respectively).

**Figure 6:**
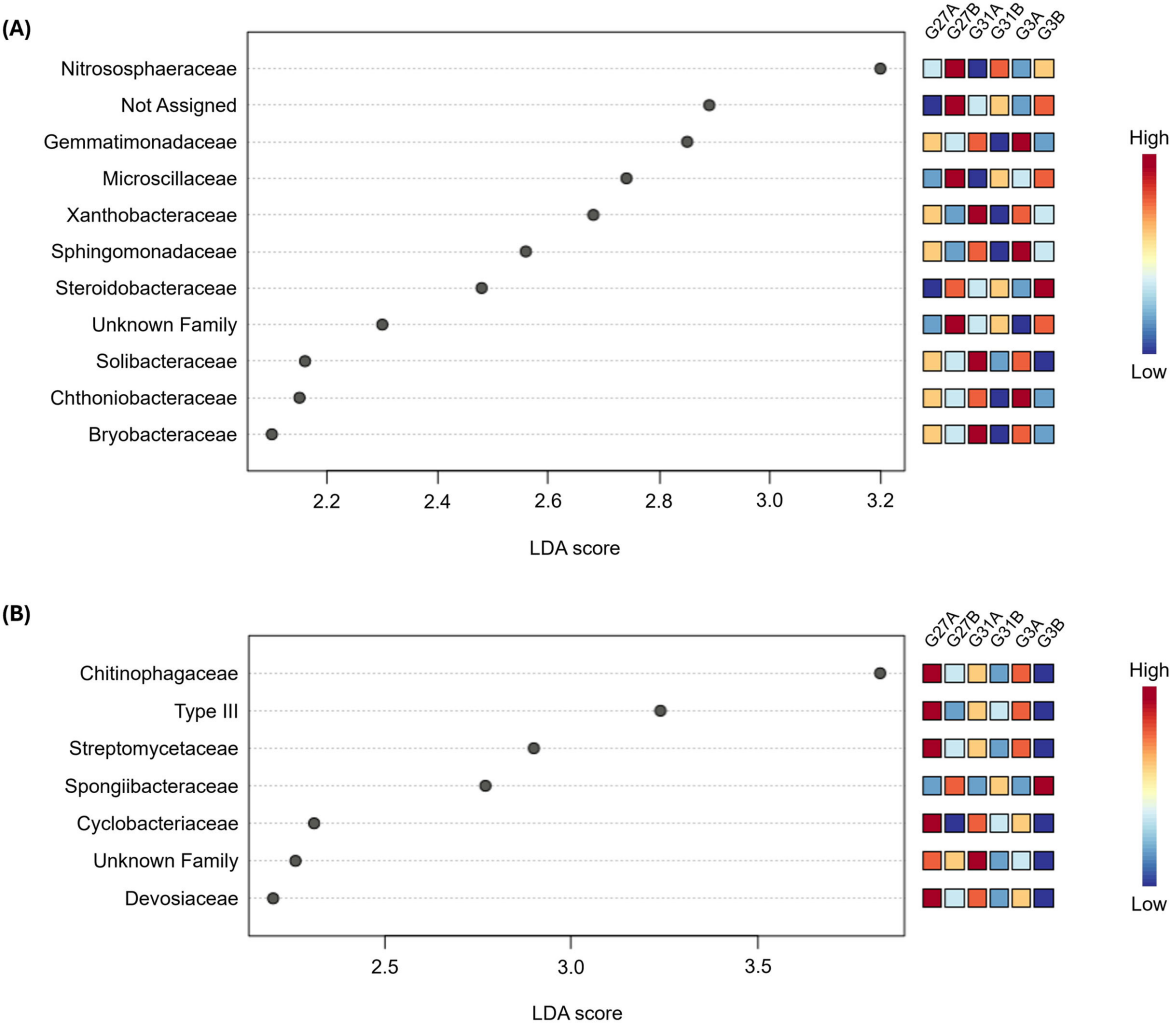
Linear discriminant analysis Effect Size (LEfSe) at the “family” level from rhizosphere soil **(A)** and roots **(B)** across the six conditions (genotype×soil) studied. Differential abundance was determined with Linear Discriminant Analysis (LDA) of Cumulative Sum Scaling (CSS) normalized counts. LDA scores are displayed by the gray dots. Families in low and high abundance are shown in blue and red squares on the right, respectively.

To further identify bacterial communities at the family level that were significantly differentially abundant across the six “genotype×soil” interactions (LDA score > 2, FDR-adjusted p < 0.05), we conducted LEfSe analysis (**Figure 6**).

In the rhizosphere soil, eleven bacterial families were identified, including one unclassified and one unassigned family (**Figure 6A**). Among these, ‘*Nitrososphaeraceae’* contributed the most to the differentiation between conditions (LDA=3.2, FDR-adjusted p = 0.017), whereas ‘*Bryobacteraceae’* contributed the least (LDA=2.1, FDR-adjusted p = 0.017) (**Supplementary Table S3**). Most identified families were significantly enriched in soil A, except for ‘*Nitrososphaeraceae’*, ‘*Microscillaceae’*, ‘*Steroidobacteraceae’*, as well as the unclassified and unassigned families. In the root compartment, seven bacterial families were identified, including one unclassified family (**Figure 6B**). Among these, ‘*Chitinophagaceae’* had the greatest contribution to the differentiation between conditions (LDA=3.83, FDR-adjusted p = 0.034), whereas ‘*Devosiaceae’* contributed the least (LDA=2.2, FDR-adjusted p = 0.049) (**Supplementary Table S4**). Like those in the rhizosphere, most families were significantly more represented in soil A, except for ‘*Spongiibacteraceae*’. Conversely, in the leaf compartment, no bacterial family emerged as significantly differentially abundant between conditions in this analysis, suggesting a more conserved endophytic phyllosphere bacterial composition. Overall, depending on the compartment (rhizosphere soil or roots) and soil type (A or B), when a given family was over- or underrepresented, this pattern remained consistent across all three genotypes. For example, in rhizosphere soil, the ‘*Nitrososphaeraceae’* family was more abundant in genotype 27 grown in soil B than in soil A; this trend was also observed for the other two genotypes. Moreover, hop genotypes exhibited varying degrees of bacterial recruitment, with no single genotype consistently acting as a ‘low’ or ‘high’ recruiter across all bacterial families. These findings suggest that hop bacterial recruitment is influenced by a complex interplay between bacterial families, hop genotypes, and soil types, as no clear overarching trend emerged from the analysis.

### 3.6 Determination of the core microbiota in the hop compartment

We investigated the core bacterial microbiota in the rhizosphere soil, roots, and leaves. In the rhizosphere soil, G3A, G27A, and G31A presented 208 (4.01%), 202 (3.89%), and 209 (4.02%) ASVs, respectively, with their corresponding G3B, G27B, and G31B conditions, representing 64, 65, and 66 bacterial families, respectively. Among them, 51 families were common across all the conditions, the most abundant of which included ‘*Bacillaceae*’, ‘*Sphingomonadaceae*’, ‘*Chitinophagaceae*’, ‘*Gemmatimonadaceae*’, ‘*Comamonadaceae*’, ‘*Pedosphaeraceae*’, ‘*Xanthobacteraceae*’, ‘*Haliangiaceae*’, ‘*Nitrososphaeraceae*’, and ‘*Planococcaceae*’ (**Supplementary Figure S5A**). In the root compartment, the number of shared ASVs was lower, with 78 (4.14%), 103 (5.47%), and 117 (6.21%) ASVs identified for G3A-G3B, G27A-G27B, and G31A-G31B, respectively, representing 32, 37, and 33 bacterial families. Among them, 25 were common across all conditions, including the most abundant: ‘*Comamonadaceae*’, ‘*Chitinophagaceae*’, ‘*type III*’ (*Entomoplasmatales* order), ‘*Caulobacteraceae*’, ‘*Microscillaceae*’, ‘*Cytophagaceae*’, ‘*env. OPS17*’ (*Sphingobacteriales* order), ‘*Crocinitomicaceae*’, ‘*Sphingomonadaceae*’, and an ‘*Unknown Family*’ (**Supplementary Figure S5B**). Unlike the other compartments, the leaf microbiota presented no consistently shared taxonomic families. However, across the rhizosphere soil and root compartments, 17 bacterial families were consistently found across all the genotypes and soil types, suggesting that these bacteria belong to the core microbiota. These included ‘*211ds20*’ (*Pseudomonadales* order), ‘*Caulobacteraceae’*, ‘*Cellvibrionaceae’*, ‘*Chitinophagaceae’*, ‘*Comamonadaceae’*, ‘*env. OPS17*’ (*Sphingobacteriales* order), ‘*Flavobacteriaceae’*, ‘*Hyphomicrobiaceae’*, ‘*Microscillaceae’*, ‘*Pseudohongiellaceae’*, ‘*Sphingomonadaceae’*, ‘*Steroidobacteraceae’*, ‘*Streptomycetaceae’*, ‘*type III*’ (*Entomoplasmatales* order), an ‘*Unknown Family’*, ‘*Xanthobacteraceae’*, and ‘*Xanthomonadaceae’* (**Supplementary Figure S5**).

### 3.7 Microbiome–metabolome interplay in wild hops

**Figure 7:**
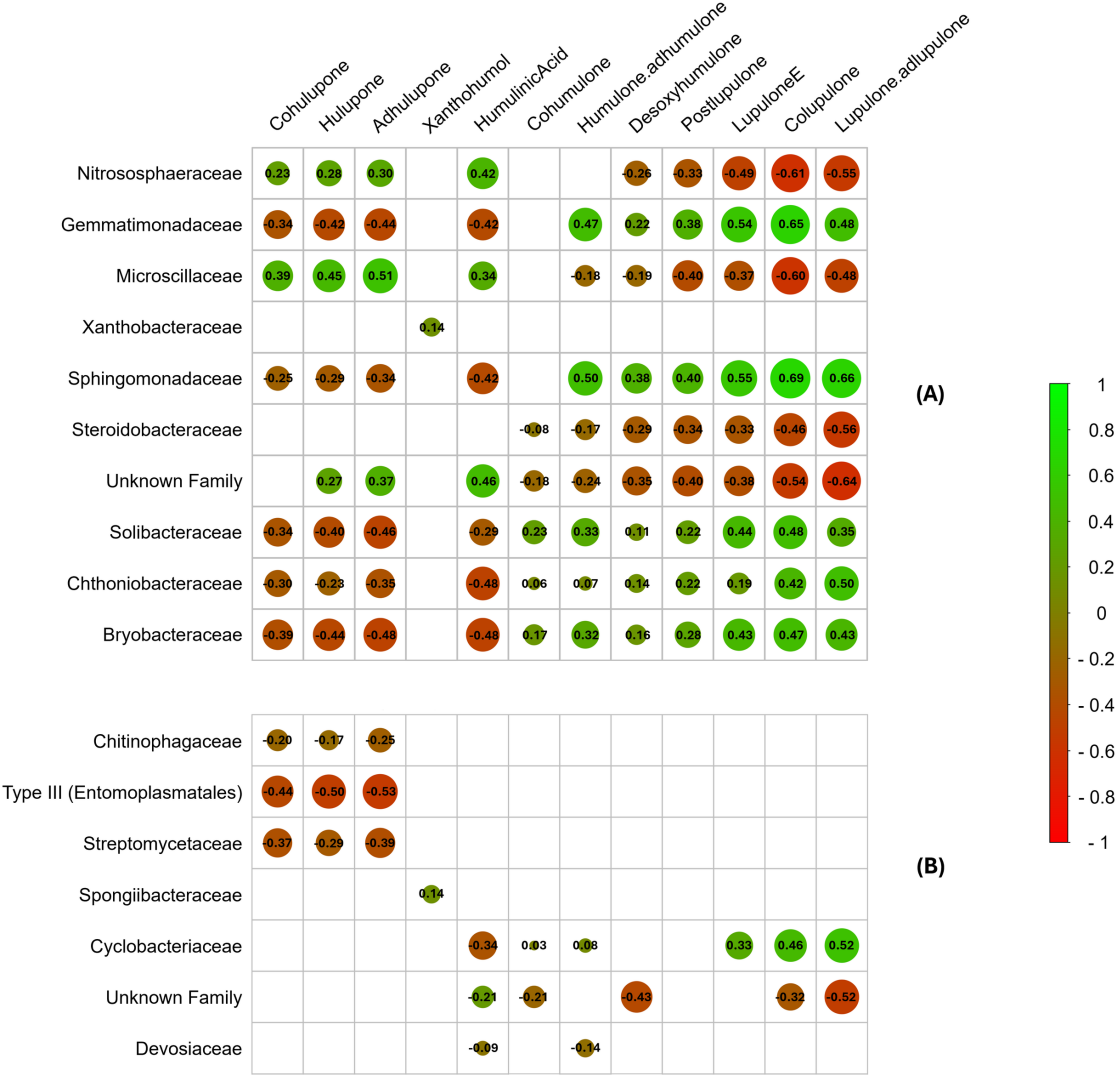
Correlation plot between specialized metabolites identified and significantly differentially abundant bacterial families in rhizosphere soil **(A)** and roots **(B)**. Spearman’s correlation coefficient is represented by the color gradient, circle size, and values in boxes. Nonsignificant correlations (p > 0.001) are indicated with blank boxes. The order of the unnamed bacterial families is indicated in parentheses.

Because no bacterial family was identified as significantly differentially abundant in the leaf compartment through LEfSe analysis, we correlated the leaf metabolic data with the bacterial families that showed significant differential abundance in the rhizosphere soil and root compartments (**Figure 7**).

Only highly significant correlations (*p*<0.001) were considered for this analysis. The strongest correlations between metabolic and metabarcoding data were observed in the rhizosphere soil compartment, although some bacterial families present in the roots were also correlated with specialized metabolites (**Figure 7**). Notably, several bacterial families were positively correlated with the synthesis of α- and β-type bitter acids (co, n-, and ad-humulone; co, n-, and ad-lupulone), which are highly valued in the brewing industry, particularly α-acids responsible for beer bitterness. These families included ‘*Gemmatimonadaceae*’, ‘*Sphingomonadaceae*’, ‘*Solibacteraceae*’, ‘*Chthoniobacteraceae*’, and ‘*Bryobacteraceae*’ in the rhizosphere (**Figure 7A**), as well as ‘*Cyclobacteriaceae*’ in the root compartment (**Figure 7B**). Conversely, other bacterial families, such as ‘*Nitrososphaeraceae*’, ‘*Microscillaceae*’, ‘*Steroidobacteraceae*’, and an unidentified family in the rhizosphere soil, presented negative correlations with the synthesis of these compounds. In contrast, xanthohumol biosynthesis appeared largely unaffected by the associated bacterial microbiota, and certain bacterial families, including ‘*Xanthobacteraceae*’ (one correlation) in the rhizosphere and ‘*Spongiibacteraceae’* and ‘*Devosiaceae*’ in the root compartment (one and two correlations, respectively), presented minimal significant correlations with specialized metabolite production. These findings suggest that specialized hop metabolite biosynthesis could be positively or negatively influenced depending on the associated bacterial microbiota. Alternatively, specialized metabolites could actively shape the recruitment and abundance of microbial communities within these compartments. Furthermore, this bacterial microbial importance in hop-specific metabolite regulation appears to be more pronounced in the rhizosphere soil than in the roots. Notably, some of the significantly differentially abundant bacterial families observed in LEfSe (**Figure 6**), such as ‘*Chitinophagaceae’*, ‘*Microscillaceae’*, ‘*Sphingomonadaceae’*, ‘*Steroidobacteraceae’*, ‘*Streptomycetaceae’*, ‘*Type III*’, ‘*Xanthobacteraceae’*, and an unidentified family, were also part of the core microbiota. However, most of these families showed either no correlation or a negative correlation with bitter acid production, with the exception of the ‘*Sphingomonadaceae*’ family.

## 4 Discussion

In this study, we investigated the influence of hop genotype and soil type on the production of specialized metabolites and the composition of bacterial communities associated with different compartments (bulk soil, rhizosphere soil, roots, and leaves) in *Humulus lupulus* L. wild germplasms.

To assess the specialized metabolite content, we conducted a chemotyping approach based on the metabolic composition of hop leaves. Our analysis identified key hop metabolites, including prenylated chalcones (xanthohumol), α- (co, n-, ad-humulone) and β- (co, n-, ad-lupulone) types of bitter acids, as well as their precursors, derivatives, and oxidized derivatives. The six ‘genotype×soil’ tested conditions presented distinct specialized metabolite profiles, driven by quantitative differences. Moreover, our results revealed that both the soil type and hop genotype significantly influenced the production of specialized metabolites in leaves, with each factor explaining approximately 28% of the observed variance. The impact of genotype on the composition of specialized hop metabolites has already been reported. For example, in a recent study, Ducrocq and collaborators demonstrated that wild hop accessions cultivated under homogeneous pedoclimatic conditions presented distinct metabolic profiles, highlighting the significant impact of hop genotype on the biosynthesis of specialized hop metabolites (Ducrocq et al., 2025). In parallel, the role of environmental factors has also been investigated (De Keukeleire et al., 2007; Van Holle et al., 2021; Rosa et al., 2025), without specifically disentangling the contribution of the ‘soil’ factor alone to the accumulation of specialized metabolites. Interestingly, the strongest effect was observed for the interaction between genotype and soil (‘genotype×soil’), which explained approximately 66% of the observed variance in specialized metabolite production. This finding highlights the complexity of metabolic regulation in hops and suggests the presence of metabolic plasticity across genotypes. Hop breeding programs aim to develop cultivars with desirable brewing properties; our results indicate that the chemical composition of a given genotype is also modulated by the soil in which it is cultivated. This ‘genotype×environment’ interaction has been previously documented in hop varieties. For example, the ‘Comet’ variety presented distinct chemical profiles in terms of bitter acid and essential oil composition depending on the cultivation location (Rosa et al., 2025). Such variations are commonly attributed to the ‘terroir’ effect, which Seguin (1986) defined as the complex interplay of environmental factors, including temperature, light availability, water management, and soil characteristics (Seguin, 1986). However, in our study, hops were grown in a greenhouse under controlled conditions, without environmental variability across conditions. This approach allowed us to isolate the specific contributions of ‘genotype’ and ‘soil’ factors and not fully consider the broader ‘terroir’ effect, as observed in field-grown cultivars (Van Holle et al., 2017; Rosa et al., 2025). Consistent with our initial hypothesis, we confirmed that both ‘genotype’ and ‘soil’ factors independently influence the accumulation of specialized metabolites in hop leaves. However, neither factor alone exerted a dominant effect. Instead, the strongest influence was observed for the interaction ‘*genotype×soil’*, which explained a much larger share of the variance (~66%), underscoring the importance of genotype*×*environment interplay in shaping hop metabolic profiles.

This pronounced ‘genotype×soil’ interaction may be driven by differences in soil microbial communities, an influence previously highlighted in *Cannabis sativa* L., a closely related species to *H. lupulus* (Ahmed and Hijri, 2021). To further investigate this potential microbiota-driven modulation, we performed a metabarcoding analysis to study the bacterial microbiota associated with different hop compartments (rhizosphere soil, roots and leaves), as well as with the bulk soil. Our results highlight a strong filtering effect, as evidenced by the decrease in α diversity richness indices. The highest bacterial richness was detected in the bulk soil, with a progressive decrease in richness from the rhizosphere soil and roots to the leaves, reflecting a progressive bacterial filtering process as we moved closer to the plant-associated compartments throughout the study (from the rhizosphere soil to the leaves). This trend aligns with a previous report in which the bacterial filtering process has already been observed in other species, such as *Cannabis sativa* (Wei et al., 2021), *Broussonetia papyrifera* (Chen et al., 2020) and *Glycine max* (Wang et al., 2022), as microbial communities become progressively less diverse in plant-associated compartments. Compared with the Chao1 index reported for *Cannabis sativa*, we observed greater bacterial richness in rhizosphere soil but lower richness in the root and leaf compartments (Wei et al., 2021). These results indicate that bacterial richness is influenced by both the plant species and the specific plant compartment studied. This phenomenon was expected, as plants select specific bacteria in the rhizosphere soil from the bulk soil’s native bacterial communities (Berendsen et al., 2012). In fact, to shape their rhizosphere soil microbiome, plants release a large variety of chemical compounds that serve as carbon and nutrient sources for microbial metabolism. These exudates, derived from photosynthetically fixed carbon (accounting for approximately 5–30% of the total fixed carbon), primarily consist of sugars, organic acids, amino acids (Glick, 2014), and specialized metabolites (Badri and Vivanco, 2009). However, plant tissue communities are shaped by more selective pressures, including the host immune response and metabolic composition (Compant et al., 2025). Moreover, our findings revealed that hop-associated bacterial communities are structured according to the compartment studied, thereby supporting our hypothesis of a distinct and specific bacterial community in the fourth compartment studied. The bacterial communities of the bulk soil and rhizosphere soil samples appeared highly similar, whereas the root- and leaf-associated communities were markedly distinct. These results align with previous findings in *Agave* species (Coleman-Derr et al., 2016) and *Cannabis sativa* (Barnett et al., 2020), where the authors observed a strongly structured microbiota according to the studied plant compartment. Contrary to our initial hypotheses that soil primarily influences external compartments (bulk and rhizosphere soils), whereas hop genotypes shape internal compartments (roots and leaves), our results showed that the bacterial communities in the three hop compartments were shaped primarily by the interaction between genotype and soil (‘genotype×soil’). In all compartments, the ‘genotype×soil’ interaction explained the largest proportion of variance (highest R², **Table 2**). In rhizosphere soil and roots, this was followed by a stronger individual effect of ‘soil’ than ‘genotype’, whereas the opposite trend was observed in leaves, where ‘genotype’ had a greater influence than the ‘soil’ factor. Interestingly, this strong ‘genotype×soil’ interaction was not observed in the root microbiota of *Lotus japonicus*, where soil had a predominant effect (R² ~22%), whereas genotype and its interaction with soil contributed similarly (R² ~4%) (Bamba et al., 2024). These findings suggest that the influence of the host plant on bacterial recruitment varies across plant species and plant compartments. Additionally, as demonstrated in this study, hops are associated with a highly diverse bacterial community in the rhizosphere soil, which is progressively selected in the roots and even more so in the leaves. Similar to the hop flower microbiota, we observed a high abundance of Proteobacteria in the leaf compartment (Allen et al., 2019). By comparing the three genotypes and hop compartments, we identified a core microbiota composed of 17 bacterial families. Interestingly, ‘*Cellvibrionaceae*’ and ‘*Xanthomonadaceae*’ were also detected in the core root microbiota of *Cannabis sativa* (Winston et al., 2014), suggesting the existence of a conserved set of bacterial families shared among closely related plant species. Furthermore, our findings revealed correlations between specific bacterial families and the biosynthesis of specialized hop metabolites, supporting our final hypothesis, which suggests that specific bacterial taxa are linked to the accumulation of specialized metabolites in leaves. Notably, families such as ‘*Gemmatimonadaceae*’ and ‘*Sphingomonadaceae*’ were positively associated with bitter acid biosynthesis, suggesting a potential role of hop-associated bacterial microbiota in modulating hop metabolism. However, it is important to note here that specialized metabolites may also influence the abundance of specific bacterial taxa, suggesting a likely bidirectional interaction between hop metabolites and associated microbiota. These results are consistent with those of previous studies. For example, in *Cannabis*, cannabinoid biosynthesis is increased when plants are associated with plant growth-promoting rhizobacteria (Pagnani et al., 2018). However, our results also indicate that xanthohumol biosynthesis is largely unaffected by bacterial communities, with only weak correlations detected. These findings suggest that several metabolic pathways may be more dependent on intrinsic genetic regulation rather than microbial influence. To further investigate the relationship between the hop microbiome and metabolome, the use of synthetic microbial communities (SynCom) could help validate the potential correlations observed in this study and provide a clearer understanding of the interactions between specialized hop metabolites and their associated microbiota. For example, Jia et al. (2024) demonstrated enhanced biosynthesis of medicinal compounds in *Salvia miltiorrhiza* when associated with a specific SynCom composed of endophytic fungi (Jia et al., 2024). However, it is important to acknowledge certain limitations of our study. First, microbiota and metabolome analyses were conducted at the vegetative stage, whereas the plant developmental stage significantly influences the microbial community composition (Comeau et al., 2020) and possibly plant metabolism. Second, the relatively short cultivation period (two months) may have limited the dynamic interactions between host plant and its associated microbiota, meaning that our findings should be interpreted as a snapshot of early-stage interactions rather than long-term trends. Third, restricting soil sampling to the uppermost layer (top 20cm), due to technical limits, may have constrained the representativeness of the original soil microbiota introduced into our experimental pots. Future studies should integrate a temporal dimension to assess hop microbiota and hop metabolism dynamics across different phenological stages. Second, our analysis focused exclusively on bacterial communities. Expanding the study to include fungal communities would provide a more comprehensive understanding of the hop holobiont. Finally, functional validation is needed to confirm the observed correlations between bacterial families and specialized metabolite biosynthesis. A metatranscriptomic approach, coupled with targeted metabolomics, could be instrumental in validating or refuting our findings.

## 5 Conclusion

To our knowledge, this study provides the first comprehensive analysis of the bacterial microbiota associated with *H. lupulus* wild germplasms from the rhizosphere soil to the phyllosphere while integrating metabolomic data. Our findings reveal that soil type, hop genotype, and their interaction significantly shape hop-associated bacterial communities, with a predominant interaction effect in each compartment (rhizosphere soil, roots and leaves). Additionally, we demonstrate that hop metabolic composition is influenced by both soil and genotype factors, as well as their interaction. Importantly, we identified potential positive correlations between specific bacterial families and specialized metabolite biosynthesis, suggesting that microbes contribute to secondary metabolism. These findings provide new perspectives for leveraging the microbiota to increase hop metabolite production, either through microbiome manipulation or targeted microbial inoculation, especially for bitter acids, which are valorized in the brewing industry. Future research should focus on the experimental validation of these interactions and explore how microbiota can be harnessed to optimize hop metabolite production for both brewing and/or health applications.

## Data Availability

All acquired raw sequencing data has been submitted in the Recherche Data Gouv database following the persistent link: https://doi.org/10.57745/CBSTP0. The corresponding author will provide any further relevant data upon reasonable request.

## References

[B1] Abdul Khalil H. P. S. Hossain M. Rosamah E. Azli N. A. Saddon N. Davoudpoura Y.. (2015). The role of soil properties and it’s interaction towards quality plant fiber: A review. Renewable Sustain. Energy Rev. 43, 1006–1015. doi: 10.1016/j.rser.2014.11.099

[B2] Ahmed B. Hijri M. (2021). Potential impacts of soil microbiota manipulation on secondary metabolites production in cannabis. J. Cannabis Res. 3, 25. doi: 10.1186/s42238-021-00082-0, PMID: 34217364 PMC8254954

[B3] Allen M. E. Piefer A. J. Cole S. N. Werner J. J. Benziger P. T. Grieneisen L.. (2019). Characterization of microbial communities populating the inflorescences of *Humulus lupulus* L. J. Am. Soc. Brew Chem. 77, 243–250. doi: 10.1080/03610470.2019.1667739

[B4] Badri D. V. Vivanco J. M. (2009). Regulation and function of root exudates. Plant Cell Environ. 32, 666–681. doi: 10.1111/j.1365-3040.2009.01926.x 19143988

[B5] Bamba M. Akyol T. Y. Azuma Y. Quilbe J. Andersen S. U. Sato S. (2024). Synergistic effects of plant genotype and soil microbiome on growth in *Lotus japonicus* . FEMS Microbiol. Ecol. 100, fiae056. doi: 10.1093/femsec/fiae056, PMID: 38678008 PMC11068475

[B6] Barnett S. E. Cala A. R. Hansen J. L. Crawford J. Viands D. R. Smart L. B.. (2020). Evaluating the microbiome of Hemp. Phytobiomes J. 4, 351–363. doi: 10.1094/PBIOMES-06-20-0046-R

[B7] Berendsen R. L. Pieterse C. M. J. Bakker P. A. H. M. (2012). The rhizosphere microbiome and plant health. Trends Plant Sci. 17, 478–486. doi: 10.1016/j.tplants.2012.04.001, PMID: 22564542

[B8] Brown S. P. Grillo M. A. Podowski J. C. Heath K. D. (2020). Soil origin and plant genotype structure distinct microbiome compartments in the model legume Medicago truncatula. Microbiome 8, 139. doi: 10.1186/s40168-020-00915-9, PMID: 32988416 PMC7523075

[B9] Bulgarelli D. Schlaeppi K. Spaepen S. Van Themaat E. V. L. Schulze-Lefert P. (2013). Structure and functions of the bacterial microbiota of plants. Annu. Rev. Plant Biol. 64, 807–838. doi: 10.1146/annurev-arplant-050312-120106, PMID: 23373698

[B10] Callahan B. J. McMurdie P. J. Rosen M. J. Han A. W. Johnson A. J. A. Holmes S. P. (2016). DADA2: High-resolution sample inference from Illumina amplicon data. Nat. Methods 13, 581–583. doi: 10.1038/nmeth.3869, PMID: 27214047 PMC4927377

[B11] Chen P. Zhao M. Tang F. Hu Y. Peng X. Shen S. (2020). The effect of plant compartments on the Broussonetia papyrifera-associated fungal and bacterial communities. Appl. Microbiol. Biotechnol. 104, 3627–3641. doi: 10.1007/s00253-020-10466-6, PMID: 32078018

[B12] Chong J. Liu P. Zhou G. Xia J. (2020). Using MicrobiomeAnalyst for comprehensive statistical, functional, and meta-analysis of microbiome data. Nat. Protoc. 15, 799–821. doi: 10.1038/s41596-019-0264-1, PMID: 31942082

[B13] Coleman-Derr D. Desgarennes D. Fonseca-Garcia C. Gross S. Clingenpeel S. Woyke T.. (2016). Plant compartment and biogeography affect microbiome composition in cultivated and native *Agave* species. New Phytol. 209, 798–811. doi: 10.1111/nph.13697, PMID: 26467257 PMC5057366

[B14] Comeau D. Novinscak A. Joly D. L. Filion M. (2020). Spatio-temporal and cultivar-dependent variations in the cannabis microbiome. Front. Microbiol. 11. doi: 10.3389/fmicb.2020.00491, PMID: 32265895 PMC7105690

[B15] Compant S. Cassan F. Kostić T. Johnson L. Brader G. Trognitz F.. (2025). Harnessing the plant microbiome for sustainable crop production. Nat. Rev. Microbiol. 23, 9–23. doi: 10.1038/s41579-024-01079-1, PMID: 39147829

[B16] Custódio V. Gonin M. Stabl G. Bakhoum N. Oliveira M. M. Gutjahr C.. (2022). Sculpting the soil microbiota. Plant J. 109, 508–522. doi: 10.1111/tpj.15568, PMID: 34743401

[B17] De Cooman L. Everaert E. De Keukeleire D. (1998). Quantitative analysis of hop acids, essential oils and flavonoids as a clue to the identification of hop varieties. Phytochem. Anal. 9, 145–150. doi: 10.1002/(SICI)1099-1565(199805/06)9:3<145::AID-PCA393>3.0.CO;2-K

[B18] De Keukeleire J. Janssens I. Heyerick A. Ghekiere G. Cambie J. Roldán-Ruiz I.. (2007). Relevance of organic farming and effect of climatological conditions on the formation of α-acids, β-acids, desmethylxanthohumol, and xanthohumol in hop (*Humulus lupulus* L.). J. Agric. Food Chem. 55, 61–66. doi: 10.1021/jf061647r, PMID: 17199314

[B19] Ducrocq F. Piutti S. Henychová A. Villerd J. Laflotte A. Girardeau L.. (2025). Fingerprinting and chemotyping approaches reveal a wide genetic and metabolic diversity among wild hops (*Humulus lupulus* L.). PloS One 20, e0322330. doi: 10.1371/journal.pone.0322330, PMID: 40327676 PMC12054859

[B20] Féchir M. Gallagher A. Weaver G. Roy C. Shellhammer T. H. (2023). Environmental and agronomic factors that impact the regional identity of Cascade and Mosaic^®^ hops grown in the Pacific Northwest. J. Sci. Food Agric. 103, 5802–5810. doi: 10.1002/jsfa.12655, PMID: 37129999

[B21] Glick B. R. (2014). Bacteria with ACC deaminase can promote plant growth and help to feed the world. Microbiol. Res. 169, 30–39. doi: 10.1016/j.micres.2013.09.009, PMID: 24095256

[B22] Gong T. Xin X. (2021). Phyllosphere microbiota: Community dynamics and its interaction with plant hosts. JIPB 63, 297–304. doi: 10.1111/jipb.13060, PMID: 33369158

[B23] Graves S. Piepho H.-P. Selzer L. Dorai-Raj S. (2024). multcompView: Visualizations of Paired Comparisons.

[B24] Jia H.-M. Zheng C.-W. Wu Y.-R. Wang H. Yan Z.-Y. (2024). Metabolomic approach reveals the mechanism of synthetic communities to promote high quality and high yield of medicinal plants—danshen (Salvia miltiorrhiza Bge.). Chem. Biol. Technol. Agric. 11, 120. doi: 10.1186/s40538-024-00651-4

[B25] Kassambara A. (2023). rstatix: Pipe-Friendly Framework for Basic Statistical Tests.

[B26] Kassambara A. Mundt F. (2020). factoextra: Extract and Visualize the Results of Multivariate Data Analyses.

[B27] Kunej U. Mikulič-Petkovšek M. Radišek S. Štajner N. (2020). Changes in the Phenolic Compounds of Hop (Humulus lupulus L.) Induced by Infection with Verticillium nonalfalfae, the Causal Agent of Hop Verticillium Wilt. Plants 9, 841. doi: 10.3390/plants9070841, PMID: 32635416 PMC7411879

[B28] Lê S. Josse J. Husson F. (2008). FactoMineR: an R package for multivariate analysis. J. Stat. Soft 25. doi: 10.18637/jss.v025.i01

[B29] Lu Y. Zhou G. Ewald J. Pang Z. Shiri T. Xia J. (2023). MicrobiomeAnalyst 2.0: comprehensive statistical, functional and integrative analysis of microbiome data. Nucleic Acids Res. 51, W310–W318. doi: 10.1093/nar/gkad407, PMID: 37166960 PMC10320150

[B30] McMurdie P. J. Holmes S. (2013). phyloseq: an R package for reproducible interactive analysis and graphics of microbiome census data. PloS One 8, e61217. doi: 10.1371/journal.pone.0061217, PMID: 23630581 PMC3632530

[B31] Moon K. (2020). _webr: Data and Functions for Web-Based Analysis_. R package version 0.1.5. Available online at: https://CRAN.R-project.org/package=webr.

[B32] Müller D. B. Vogel C. Bai Y. Vorholt J. A. (2016). The plant microbiota: systems-level insights and perspectives. Annu. Rev. Genet. 50, 211–234. doi: 10.1146/annurev-genet-120215-034952, PMID: 27648643

[B33] Neuwirth E. (2022). RColorBrewer: ColorBrewer Palettes.

[B34] Ogle D. Doll J. Wheeler A. Dinno A. (2025). FSA: Simple Fisheries Stock Assessment Methods.

[B35] Oksanen J. Simpson G. Blanchet F. Kindt R. Legendre P. Minchin P.. (2025). vegan: Community Ecology Package.

[B36] Pagnani G. Pellegrini M. Galieni A. D’Egidio S. Matteucci F. Ricci A.. (2018). Plant growth-promoting rhizobacteria (PGPR) in Cannabis sativa ‘Finola’ cultivation: An alternative fertilization strategy to improve plant growth and quality characteristics. Ind. Crops Prod, 75–83. doi: 10.1016/j.indcrop.2018.06.033

[B37] Pascale A. Proietti S. Pantelides I. S. Stringlis I. A. (2020). Modulation of the root microbiome by plant molecules: the basis for targeted disease suppression and plant growth promotion. Front. Plant Sci. 10. doi: 10.3389/fpls.2019.01741, PMID: 32038698 PMC6992662

[B38] Pistelli L. Ferri B. Cioni P. L. Koziara M. Agacka M. Skomra U. (2018). Aroma profile and bitter acid characterization of hop cones (Humulus lupulus L.) of five healthy and infected Polish cultivars. Ind. Crops Prod 124, 653–662. doi: 10.1016/j.indcrop.2018.08.009

[B39] Quast C. Pruesse E. Yilmaz P. Gerken J. Schweer T. Yarza P.. (2012). The SILVA ribosomal RNA gene database project: improved data processing and web-based tools. Nucleic Acids Res. 41, D590–D596. doi: 10.1093/nar/gks1219, PMID: 23193283 PMC3531112

[B40] R Core Team (2024). R: A language and environment for statistical computing. Available online at: https://www.R-project.org/.

[B41] Rosa R. S. Caetano Da Silva Lannes S. Shellhammer T. H. (2025). Analyzing terroir influence on comet hops: A comparison of chemical and sensory profiles of United States and Brazil. J. Am. Soc. Brew Chem., 1–9. doi: 10.1080/03610470.2025.2468020

[B42] Ruggeri R. Tolomio M. Muganu M. Loreti P. Virga G. Iacuzzi N.. (2023). Establishment of a commercial organic hopyard in a Mediterranean environment: Production attributes and their relationship with soil texture. Sci. Hortic. 310, 111720. doi: 10.1016/j.scienta.2022.111720

[B43] Seguin G. (1986). ‘Terroirs’ and pedology of wine growing. Experientia 42, 861–873. doi: 10.1007/BF01941763

[B44] Simon J.-C. Marchesi J. R. Mougel C. Selosse M.-A. (2019). Host-microbiota interactions: from holobiont theory to analysis. Microbiome 7, 5. doi: 10.1186/s40168-019-0619-4, PMID: 30635058 PMC6330386

[B45] Tkacz A. Cheema J. Chandra G. Grant A. Poole P. S. (2015). Stability and succession of the rhizosphere microbiota depends upon plant type and soil composition. ISME J. 9, 2349–2359. doi: 10.1038/ismej.2015.41, PMID: 25909975 PMC4611498

[B46] Van Holle A. Muylle H. Haesaert G. Naudts D. De Keukeleire D. Roldán-Ruiz I.. (2021). Relevance of hop terroir for beer flavour. J. Inst Brew 127, 238–247. doi: 10.1002/jib.648

[B47] Van Holle A. Van Landschoot A. Roldán-Ruiz I. Naudts D. De Keukeleire D. (2017). The brewing value of Amarillo hops (*Humulus lupulus* L.) grown in northwestern USA: A preliminary study of terroir significance: The Brewing Value of Amarillo Hops (*Humulus lupulus L.*) Grown in northwestern USA: A Study of Terroir Significance. J. Inst Brew 123, 312–318. doi: 10.1002/jib.433

[B48] Wang X. Wang M. Wang L. Feng H. He X. Chang S.. (2022). Whole-plant microbiome profiling reveals a novel geminivirus associated with soybean stay-green disease. Plant Biotechnol. J. 20, 2159–2173. doi: 10.1111/pbi.13896, PMID: 35869670 PMC9616524

[B49] Warnes G. Bolker B. Bonebalker L. Gentleman R. Huber W. Liaw A.. (2024). gplots: Various R Programming Tools for Plotting Data.

[B50] Wei G. Ning K. Zhang G. Yu H. Yang S. Dai F.. (2021). Compartment niche shapes the assembly and network of cannabis sativa-associated microbiome. Front. Microbiol. 12. doi: 10.3389/fmicb.2021.714993, PMID: 34675893 PMC8524047

[B51] Wei T. Simko V. (2024). corrplot: Visualization of a Correlation Matrix.

[B52] Wickham H. (2016). ggplot2: Elegant Graphics for Data Analysis (Springer).

[B53] Wickham H. Averick M. Bryan J. Chang W. McGowan L. François R.. (2019). Welcome to the tidyverse. JOSS 4, 1686. doi: 10.21105/joss.01686

[B54] Wickham H. François R. Henry L. Müller K. Vaughan D. (2023a). dplyr: A Grammar of Data Manipulation.

[B55] Wickham H. Pedersen T. Seidel D. (2023b). scales: Scale Functions for Visualization.

[B56] Wickham H. Vaughan D. Girlich M. (2024). tidyr: Tidy Messy Data.

[B57] Winston M. E. Hampton-Marcell J. ZarraonaIndia I. Owens S. M. Moreau C. S. Gilbert J. A.. (2014). Understanding cultivar-specificity and soil determinants of the cannabis microbiome. PLoS One 9, e99641. doi: 10.1371/journal.pone.0099641, PMID: 24932479 PMC4059704

[B58] Zanoli P. Zavatti M. (2008). Pharmacognostic and pharmacological profile of Humulus lupulus L. J. Ethnopharmacol 116, 383–396. doi: 10.1016/j.jep.2008.01.011, PMID: 18308492

