# Supplementary material for: Influence of genotype and soil on specialized metabolites production and bacterial microbiota associated to wild hop (Humulus lupulus L.): an early-stage study
Source: Front Plant Sci. 2025 Oct 21;16:1702956. doi: 10.3389/fpls.2025.1702956 (PMC12582967; doi:10.3389/fpls.2025.1702956)
Supplement: Supplementary file 1 [file SupplementaryFile1.zip › Supplementary File 1/Table 1.DOCX]

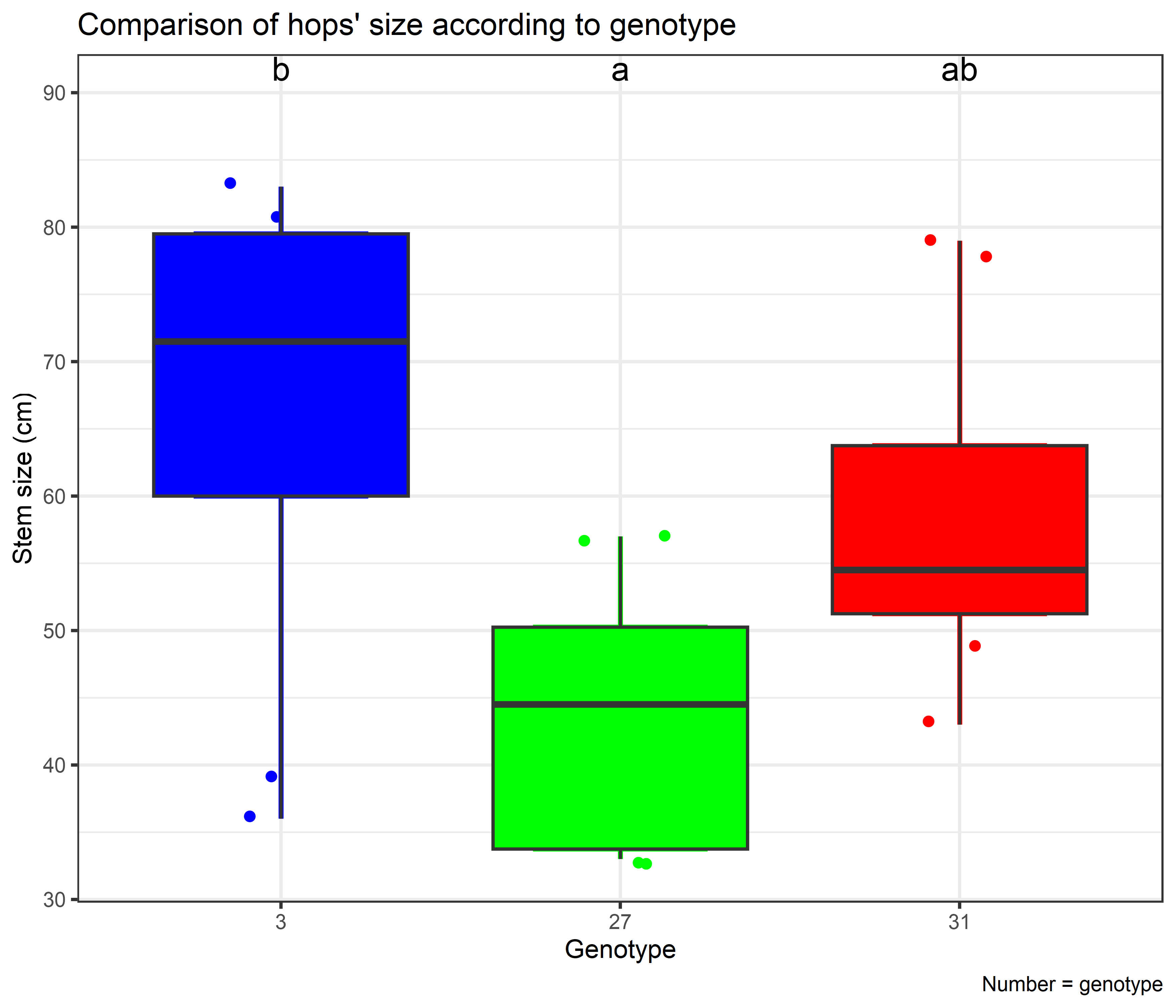
(A)


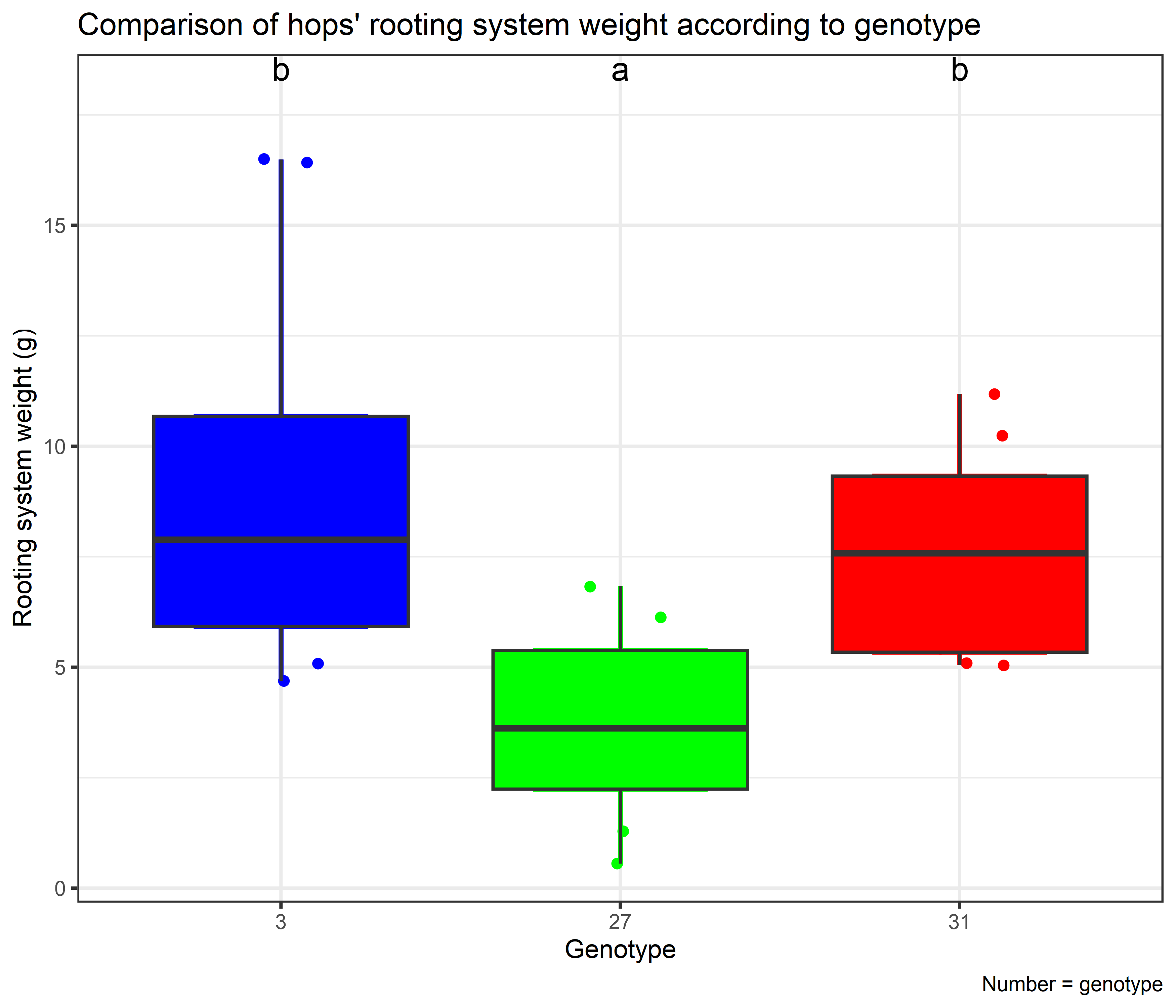
(B)

**Fig. S1** Boxplot of hop stem size (cm) **(A)** and hop fresh rooting system weight (g) **(B)**. Letters indicate statistical differences between conditions: conditions sharing the same letter are not significantly different.
