# Supplementary material for: Influence of genotype and soil on specialized metabolites production and bacterial microbiota associated to wild hop (Humulus lupulus L.): an early-stage study
Source: Front Plant Sci. 2025 Oct 21;16:1702956. doi: 10.3389/fpls.2025.1702956 (PMC12582967; doi:10.3389/fpls.2025.1702956)
Supplement: Supplementary file 1 [file SupplementaryFile1.zip › Supplementary File 1/Table 2.DOCX]

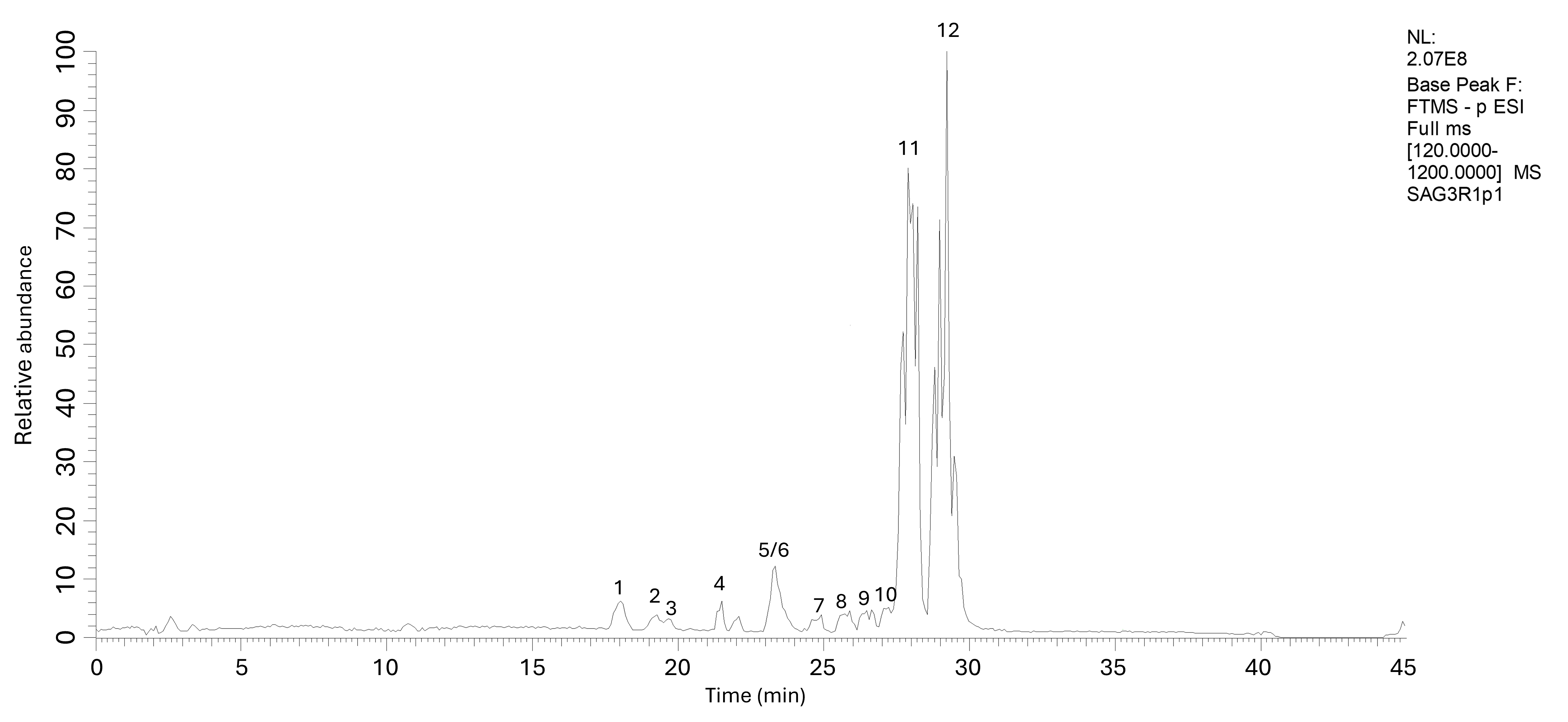


**Fig. S2** UHPLC-MS chromatogram ([M-H]^-^) from a hop leaf extract. NL: Normalization Level, FTMS: Fourier Transform Mass Spectrometry, ESI: Electrospray Ionisation. (XCalibur software - Qual Browser application, Thermo Fisher Scientific). Identified peaks are annotated from 1 to 12. Cohulupone (1), hulupone (2), adhulupone (3), xanthohumol (4), humulinic acid (5), cohumulone (6), humulone/adhumulone (7), desoxyhumulone (8), postlupulone (9), lupulone E (10), colupulone (11) and lupulone/adlupulone (12).
