# Supplementary material for: Influence of genotype and soil on specialized metabolites production and bacterial microbiota associated to wild hop (Humulus lupulus L.): an early-stage study
Source: Front Plant Sci. 2025 Oct 21;16:1702956. doi: 10.3389/fpls.2025.1702956 (PMC12582967; doi:10.3389/fpls.2025.1702956)
Supplement: Supplementary file 1 [file SupplementaryFile1.zip › Supplementary File 1/Table 4.DOCX]

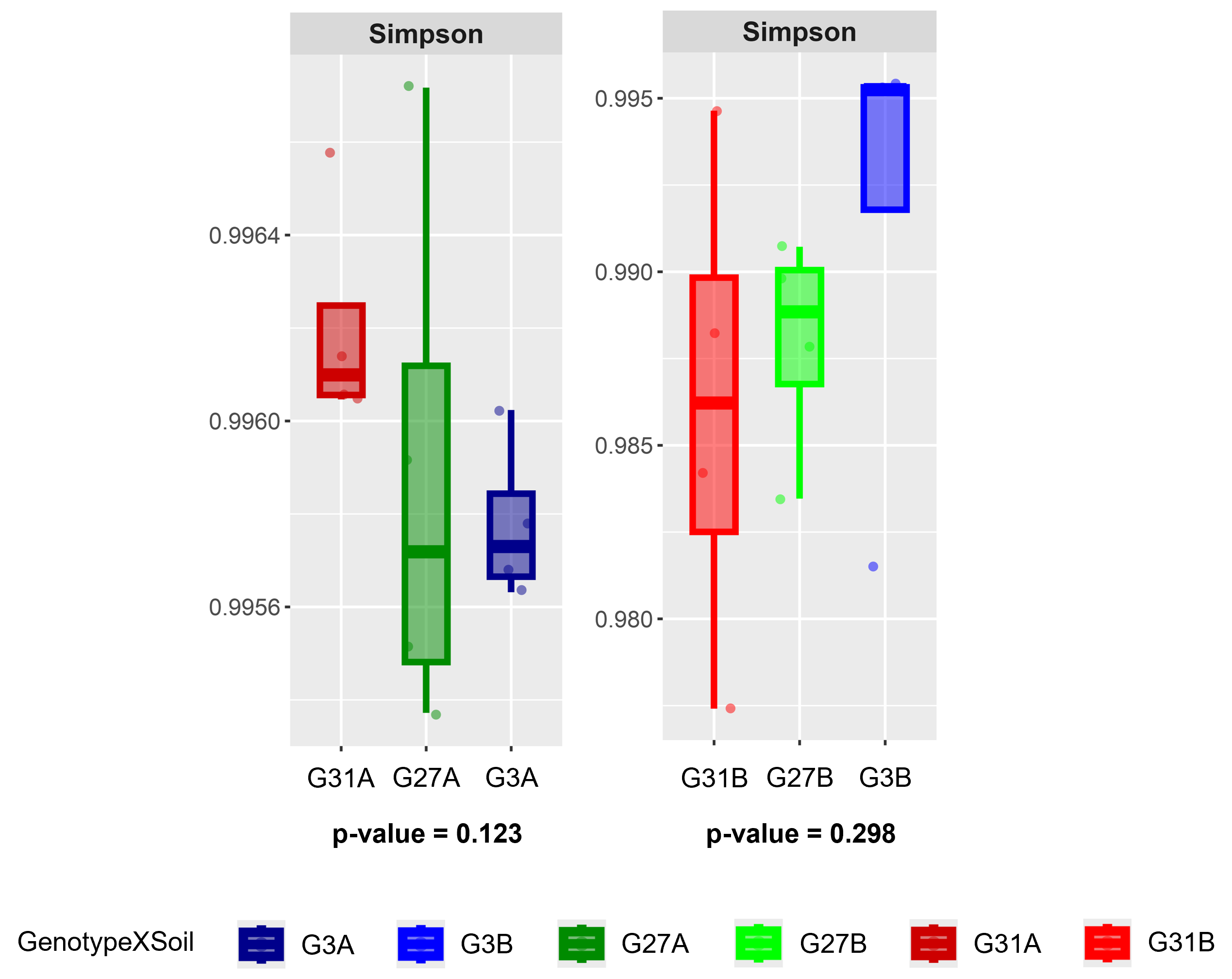
(A)


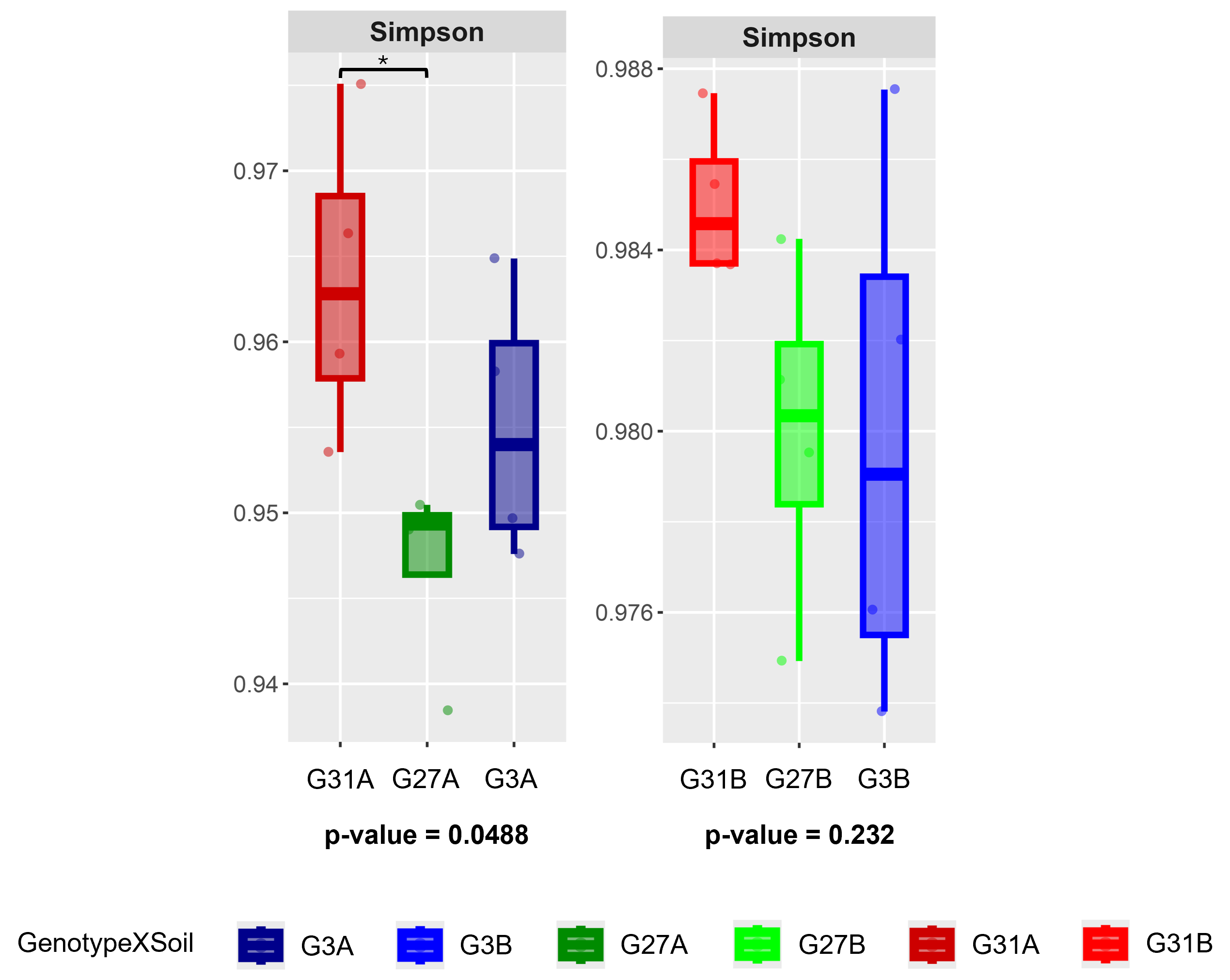
(B)


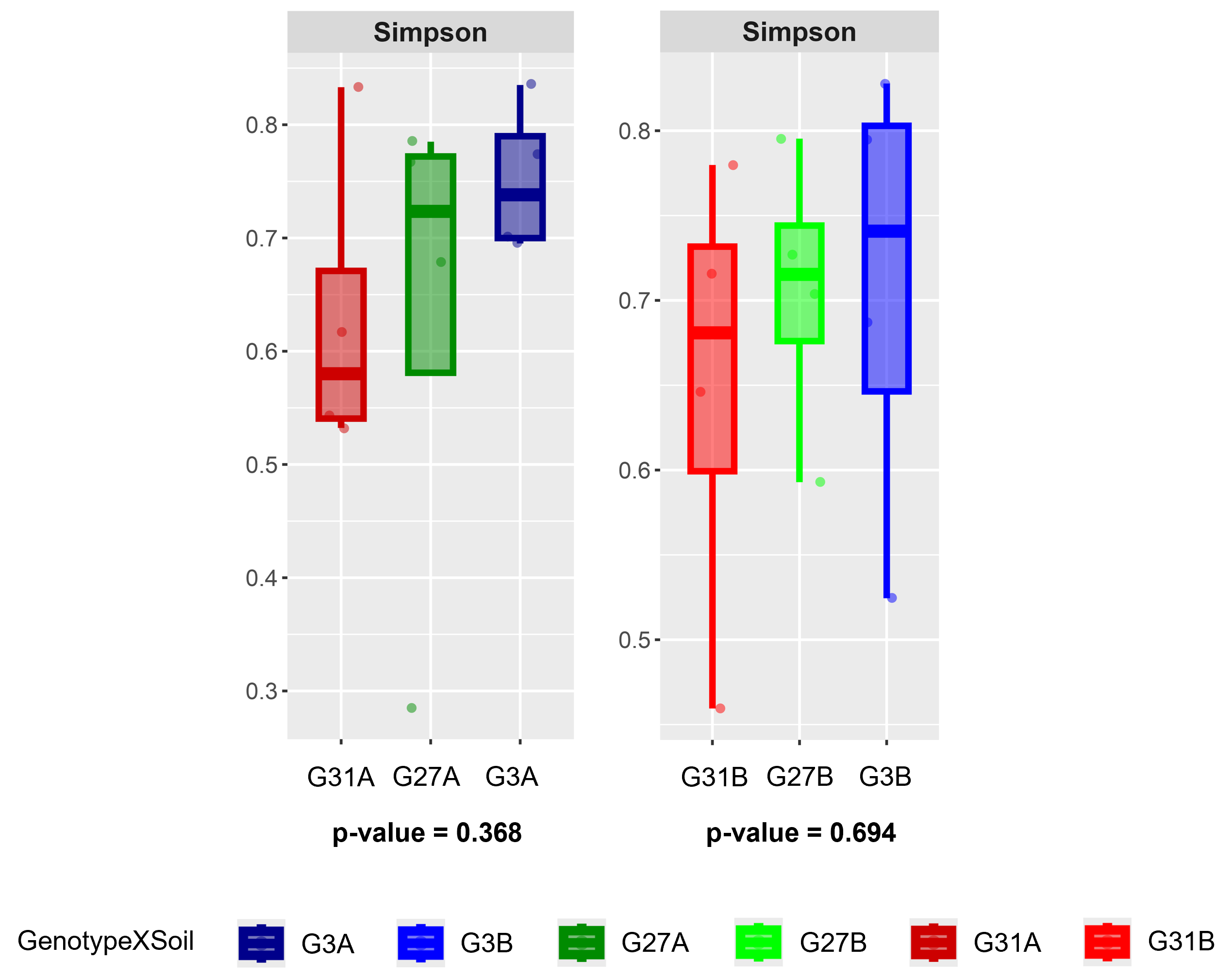
(C)

**Fig. S4** α-diversity indices across the rhizosphere soil **(A)**, root **(B)**, and leave **(C)** compartment according to ‘soil type’ subdivision. Kruskal-Wallis test p-values indicate significant differences, and (*) denotes significant differences based on post-hoc Dunn’s tests.
