# Supplementary material for: Influence of genotype and soil on specialized metabolites production and bacterial microbiota associated to wild hop (Humulus lupulus L.): an early-stage study
Source: Front Plant Sci. 2025 Oct 21;16:1702956. doi: 10.3389/fpls.2025.1702956 (PMC12582967; doi:10.3389/fpls.2025.1702956)
Supplement: Supplementary file 1 [file SupplementaryFile1.zip › Supplementary File 1/Table 5.DOCX]

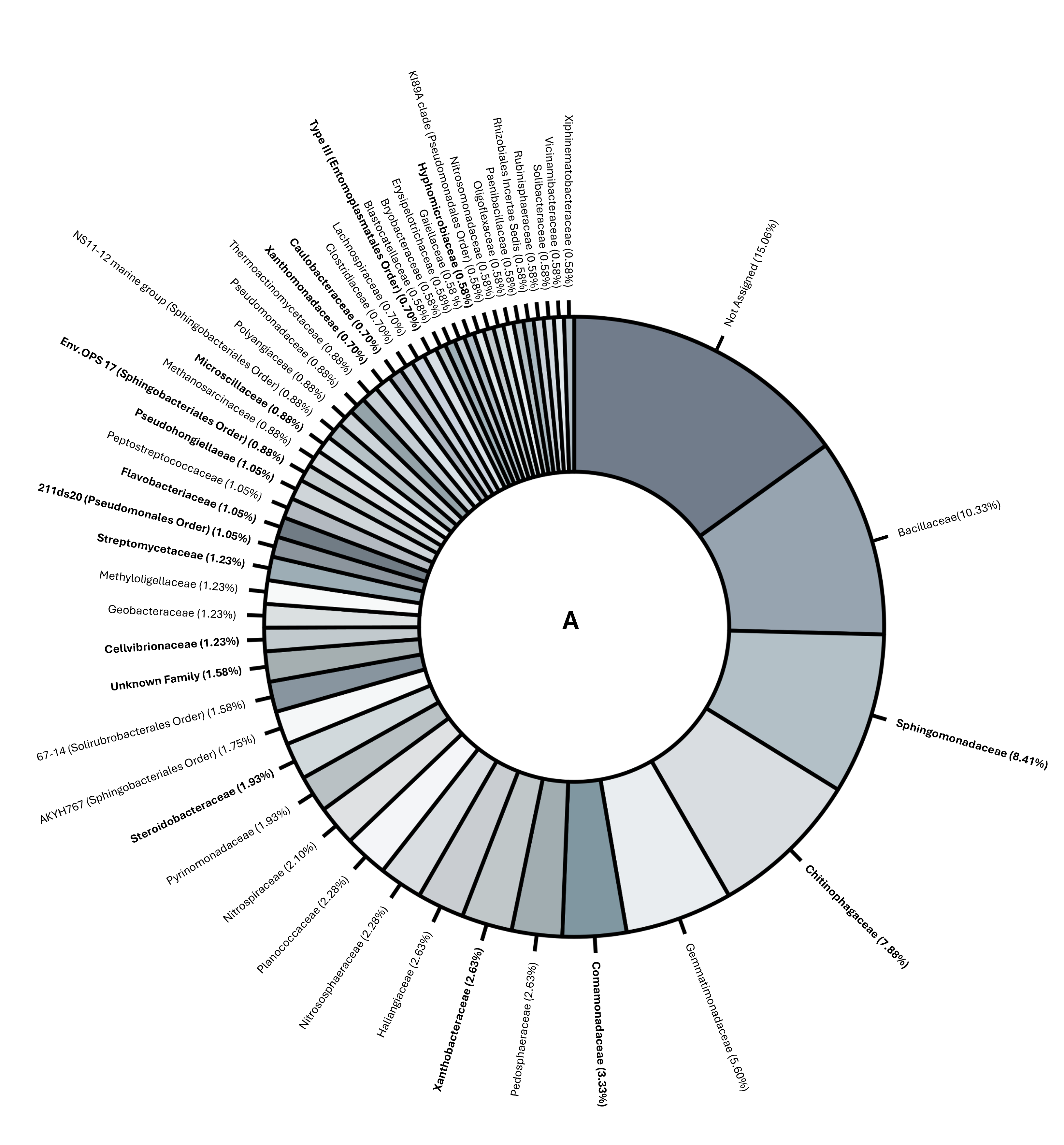


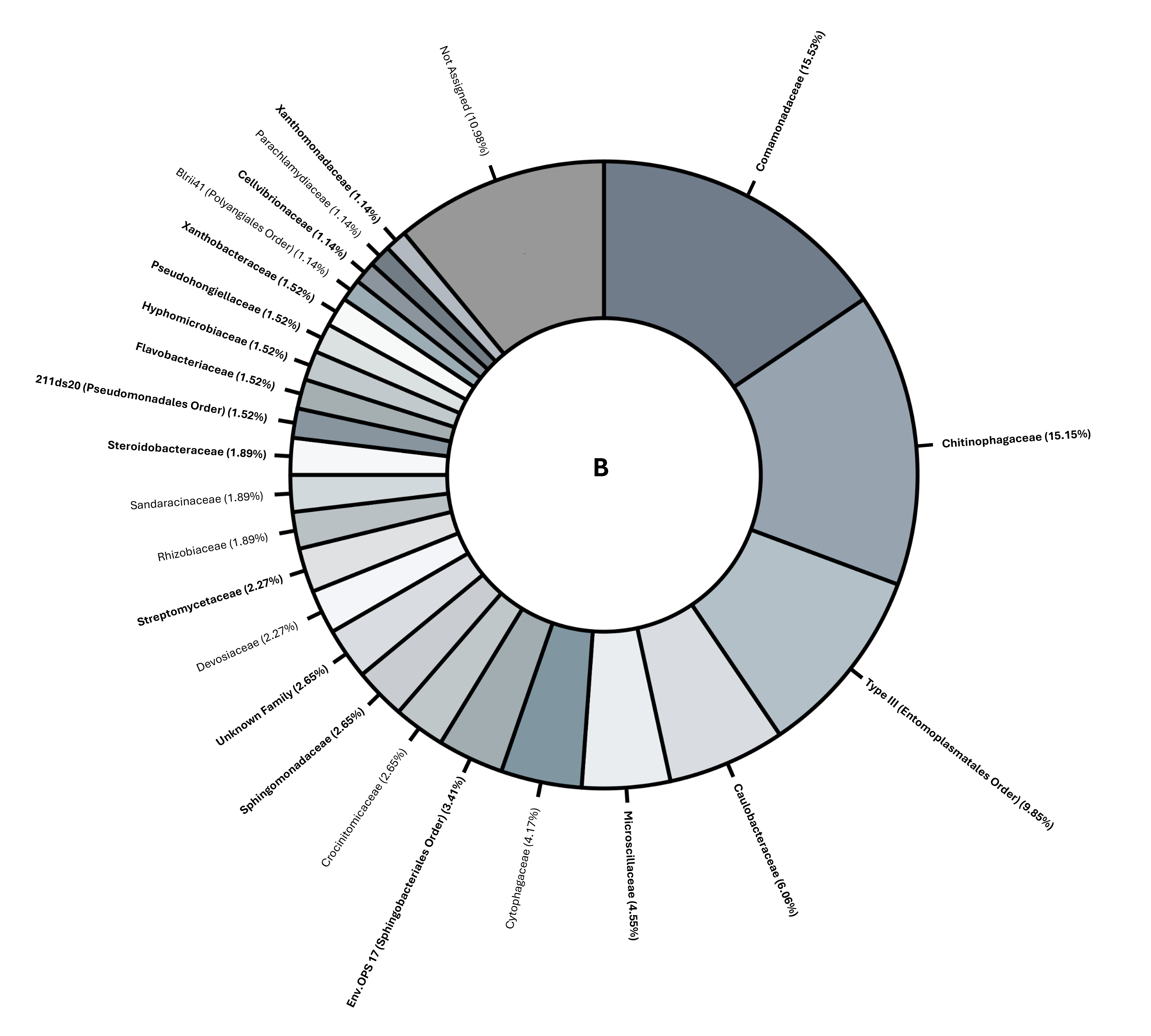


**Fig. S5** Core microbiota (bacterial families shared regardless of hop genotype and soil type) in the rhizosphere soil **(A)** and roots **(B)**. Shared bacterial families in both compartments are highlighted in bold text. For unnamed bacterial families, their order is indicated in parentheses.
